# Early Life Microbiota Colonization at Six Months of Age: A Transitional Time Point

**DOI:** 10.3389/fcimb.2021.590202

**Published:** 2021-03-26

**Authors:** Benedetta Raspini, Mirco Vacca, Debora Porri, Rachele De Giuseppe, Francesco Maria Calabrese, Marcello Chieppa, Marina Liso, Rosa Maria Cerbo, Elisa Civardi, Francesca Garofoli, Maria De Angelis, Hellas Cena

**Affiliations:** ^1^ Department of Public Health, Experimental and Forensic Medicine, Dietetics and Clinical Nutrition Laboratory, University of Pavia, Pavia, Italy; ^2^ Department of Soil, Plant and Food Science, University of Bari Aldo Moro, Bari, Italy; ^3^ National Institute of Gastroenterology “S. de Bellis”, Institute of Research, Castellana Grotte, Italy; ^4^ Neonatal Unit and Neonatal Intensive Care Unit, Fondazione IRCCS Policlinico San Matteo, Pavia, Italy; ^5^ Clinical Nutrition and Dietetics Service, Unit of Internal Medicine and Endocrinology, ICS Maugeri IRCCS, Pavia, Italy

**Keywords:** newborn, neonatal microbiota, maternal factors, breast milk, weaning, older siblings, delivery

## Abstract

**Background:**

Early life gut microbiota is involved in several biological processes, particularly metabolism, immunity, and cognitive neurodevelopment. Perturbation in the infant’s gut microbiota increases the risk for diseases in early and later life, highlighting the importance of understanding the connections between perinatal factors with early life microbial composition. The present research paper is aimed at exploring the prenatal and postnatal factors influencing the infant gut microbiota composition at six months of age.

**Methods:**

Gut microbiota of infants enrolled in the longitudinal, prospective, observational study “A.MA.MI” (*Alimentazione MAmma e bambino nei primi MIlle giorni*) was analyzed. We collected and analyzed 61 fecal samples at baseline (meconium, T0); at six months of age (T2), we collected and analyzed 53 fecal samples. Samples were grouped based on maternal and gestational weight factors, type of delivery, type of feeding, time of weaning, and presence/absence of older siblings. Alpha and beta diversities were evaluated to describe microbiota composition. Multivariate analyses were performed to understand the impact of the aforementioned factors on the infant’s microbiota composition at six months of age.

**Results:**

Different clustering hypotheses have been tested to evaluate the impact of known metadata factors on the infant microbiota. Neither maternal body mass index nor gestational weight gain was able to determine significant differences in infant microbiota composition six months of age. Concerning the type of feeding, we observed a low alpha diversity in exclusive breastfed infants; conversely, non-exclusively breastfed infants reported an overgrowth of *Ruminococcaceae* and *Flavonifractor*. Furthermore, we did not find any statistically significant difference resulting from an early introduction of solid foods (before 4 months of age). Lastly, our sample showed a higher abundance of clostridial patterns in firstborn babies when compared to infants with older siblings in the family.

**Conclusion:**

Our findings showed that, at this stage of life, there is not a single factor able to affect in a distinct way the infants’ gut microbiota development. Rather, there seems to be a complex multifactorial interaction between maternal and neonatal factors determining a unique microbial niche in the gastrointestinal tract.

## Introduction

Research on the human gut microbiome has gained attention in the past years due to the vital contribution of microorganisms to host health across the life span. Microbial colonization indeed plays a pivotal role in stimulating immune system development, nutrient metabolism, and promoting differentiation of mucosal structure and function (Kumbhare et al., 2019). The interaction between host and microbiota is essential during the first years of life because substantial shifts in the abundance and structure occur in this critical stage of the infants’ life. The intestinal microbiota undergoes dynamic development during the first years of life, determining adult microbiota composition and, consequently, impacting health. Many factors influence the shaping of gut microbiota in this critical window of plasticity, including gestational age, maternal pre-pregnancy BMI, weight gain during pregnancy, mode of birth, feeding types, weaning, birth environment, besides ethnic/geographical background (Nagpal et al., 2017; Raspini et al., 2020).

Among these factors overweight and obesity are of great concern since the number of pregnant women affected by overweight or obesity has increased both in high income and middle-income countries (Chen et al., 2018). In the US more than half of all pregnant women are affected by obesity (Flegal et al., 2012). These subjects are likely to face several complications such as gestational diabetes, hypertension, and delivery by cesarean section (Catalano and Shankar, 2017). Furthermore, it has been demonstrated that their offspring is likely to develop obesity in later life (Whitaker, 2004; Pirkola et al., 2010; Deierlein et al., 2011; Mehta et al., 2011; Gaillard et al., 2013) as well as non-communicable diseases (NCDs) (Glastras et al., 2018). These undesired effects are, at least partly, modulated by the related changes in gut microbial composition during pregnancy and lactation (Collado et al., 2012; Singh et al., 2017). Such changes impact on maternal and offspring health, altering host metabolic pathways and remodeling the expression of genes regulating them (Gaillard et al., 2013; Galley et al., 2014; Gallardo et al., 2015; Gohir et al., 2015; Kumbhare et al., 2019). Furthermore, evidence shows that breastfeeding is one of the key players in preventing alteration in gut microbiota composition, which likely contributes to the development of autoimmune and metabolic disorders later in life (Ho et al., 2018). Human milk contains remarkable bioactive compounds, including human milk oligosaccharides, HMOs, beneficial to infants as they not only promote a better growth but also strengthen the immune system of the newborn, reducing the risk of diarrhea and consequent dehydration, protecting against allergies and metabolic disorders (Ho et al., 2018). Longitudinal studies have indicated that the infant’s microbial structure varies significantly with the suspension of breast/formula feeding and, consequently, with the introduction of solid foods (Thompson et al., 2015). The weaning process represents the final path of infant gut microbial shaping, characterized by significant shifts in taxonomic groups, and the increase in gut microbial diversity, into a stable adult composition. In this process, diet plays a key role in modulating the microbial community (Stewart et al., 2018; Kumbhare et al., 2019). As solid foods are included in the diet, the microbiota starts evolving from a simple environment, Bifidobacteria-rich (microorganisms that metabolize HMOs), to a different one, rich in species such as *Bacteroides*, able to metabolize starches present in a more complex dietary pattern (Moore and Townsend, 2019). Moreover, previous studies (Flores et al., 2014; Tamburini et al., 2016) have shown that the newborns’ environment is also a natural source of germs that may colonize different body sites. For example, cohabitation boosts bacterial exchange probability from touching shared surfaces, using shared objects, and breathing indoor air (Flores et al., 2014; Tamburini et al., 2016). Other investigations, indeed, have examined the association between the presence of older siblings and increased diversity and richness of the gut microbial during early childhood, which could contribute to the substantiation of the hygiene hypothesis (Strachan, 1989; Azad et al., 2013; Laursen et al., 2015).

According to those findings, the present study explored prenatal factors, including maternal BMI and weight gain during pregnancy, as well as newborn postnatal exposure factors, including mode of feeding, time of weaning, and the presence of siblings in the family that might influence the infant gut microbiota composition at age 6 months to identify major active actors.

## Materials and Methods

### Study Design

The present study is part of the longitudinal, prospective, observational study A.MA.MI (*Alimentazione MAmma e bambino nei primi MIlle giorni*), ClinicalTrials.gov identifier: NCT04122612. The study was approved by the Human Ethics Committee (EC) of *Fondazione IRCCS Policlinico S. Matteo of Pavia* (Protocol number: 20180022618; 6/12/2018), and it was conducted on a group of mother–infant pairs referred to the Neonatal Unit, Fondazione IRCCS Policlinico San Matteo, Pavia (Italy) from birth to 1 year of age, according to the Good Clinical Practice guidelines. Written informed consent of the parents/legal guardian was provided. The Human EC of *Fondazione IRCCS Policlinico S. Matteo of Pavia* approved this procedure after ascertaining its compliance with the dictates of the Declaration of Helsinki (IV Adaptation).

The complete study design and the study protocol were previously described elsewhere (Raspini et al., 2020).

53 fecal samples were collected and analyzed at 6 months of infants’ age, corresponding to the 3^rd^ sampling of the A.MA.MI project (T2) (resuming data are presented in **Table S1**). Analyses were also performed at baseline (*meconium*, T0) on 61 samples (we did not receive eight T2 fecal samples). To evaluate the early microbiota colonization, we investigated maternal factors, such as pre-pregnancy body mass index (BMI) and gestational weight gain (WG) and perinatal factors as type of delivery, type of feeding, time of weaning, and environmental influences (due to the presence of older siblings in the household).

We collected anthropometric data as maternal height and pre-gestational weight to calculate body mass index (BMI; kg/m^2^). Based on pre-gestational BMI, women were then stratified as Normal Weight (NW—BMI ≤ 24.9 kg/m^2^) or Overweight/with Obesity (OW/OB—BMI ≥ 25 kg/m^2^) while gestational weight gain (WG) was defined as body weight increase from pre-pregnancy to delivery and compared with recommended WG ranges by IOM guidelines for each pre-pregnancy BMI category (NW, 11.5–16 kg; OW, 7–11.5 kg; and OB, 5–9 kg) (Rasmussen and Yaktine, 2009).

The samples were grouped based on maternal pre-pregnancy BMI (NW: normal pre-pregnancy BMI or OW: excessive BMI), gestational weight gain (NWG normal gestational WG or EWG: excessive gestational WG), delivery mode (VD: vaginal delivery or CS: caesarean section) type of feeding (*_E_*BF: exclusively breastfed or *_Ne_*BF: not exclusively breastfed, which include exclusively formula-fed infants and mixed fed infants), weaning (evaluating the solid food introduction if before or after 4 months of age; ≤4 or >4, respectively), and presence (nFB)/absence (FB) of older siblings.

### Samples Analysis

Stool samples were shipped on dry ice to Genomix4Life Srl (C/O Laboratory of Molecular and Genomic Medicine—Campus of Medicine and Surgery, Baronissi, Salerno, Italy, a spin-off of the University of Salerno, Fisciano, Italy) where 16S rRNA gene amplicon analysis was carried out. To ensure the personal privacy, samples had only the study ID number; no clinical or personal information was shipped.

### 16S rRNA Metagenomic Sequencing Library Preparation, Gene Amplicon Sequencing and Analysis

Next-generation sequencing (NGS) experiments, comprising DNA extraction and primary bioinformatics analysis, were performed by Genomix4life S.R.L. (Baronissi, Salerno, Italy). DNA extraction was performed with Invimag Stool kit (Stratec) using an extraction negative control. Final yield and quality of extracted DNA were determined by using NanoDrop ND-1000 spectrophotometer (Thermo Scientific, Waltham, MA) and Qubit Fluorometer 1.0 (Invitrogen Co., Carlsbad, CA). 16S rRNA gene amplification was performed with primers: Forward: 5′-CCTACGGGNGGCWGCAG-3′ and Reverse: 5′-GACTACHVGGGTATCTAATCC-3′ (Klindworth et al., 2013), which target the hypervariable V3 and V4 regions of the 16S rRNA gene. Each PCR reaction was assembled according to Metagenomic Sequencing Library Preparation (Illumina, San Diego, CA). A negative control is included in the workflow; it consists of all reagents used during sample processing (16S amplification and library preparation) but does not contain a sample to assess potential contamination. Libraries were quantified using Qubit fluorometer (Invitrogen Co., Carlsbad, CA) and pooled to an equimolar amount of each index-tagged sample to a final concentration of 4 nM, including the Phix Control Library. Pooled samples were subject to cluster generation and sequenced on MiSeq platform (Illumina, San Diego, CA) in a 2 × 300 paired-end format. The raw sequence files generated (fast files) underwent quality control analysis with FastQC. The 16S metagenomics analysis performs the taxonomic classification of 16S rRNA targeted amplicon reads after OTU clustering based on the 97% of similarity (3% of divergence). The algorithm is a high-performance implementation of the Ribosomal Database Project (RDP) Classifier described in Wang et al., 2007 (http://dx.doi.org/10.1128%2FAEM.00062-07). Taxonomic databases used to perform taxonomic classification are RefSeq RDP 16S v3 May 2018 DADA2 32bp.

The obtained sequences were uploaded to a public database, and the following extremes refer to the submission to NCBI database (http://www.ncbi.nlm.nih.gov/bioproject/675753, extremes: Submission ID: SUB8491261; BioProject ID: PRJNA675753).

### Statistical Analyses

Data were summarized using descriptive statistics, such as means and standard deviations, median, or interquartile range (IQR), as appropriate, for quantitative variables and relative frequencies for qualitative ones.

Multivariable association between 16S rRNA gene data abundances at different taxonomic levels occurring in infants’ microbiota (relative to prenatal and postnatal factors) was performed using the MaAsLin2 R package (https://huttenhower.sph.harvard.edu/maaslin/). Meanwhile, unless specifically described, data and group differences were analyzed and compared by paired or unpaired, two-tailed Student’s t-test.

To investigate the intestinal microbiota development of infants, principal component analysis (PCA) was used to evaluate the beta diversity occurring within our population to assess differences in the microbial composition [baseline (*meconium*), T0, *vs* six months of age, T2]. The dudi.pca function within the “ade4” R package (https://cran.r-project.org/web/packages/ade4/) was used to perform a PCA of data frames. The resulting PCA and dudi class objects were plotted with the “factoextra” R package (https://cran.r-project.org/web/packages/factoextra/index.html).

Looking for evidence of clustering among our samples, those genera with a median relative abundance lower than 0.1 were purged out and in first instance discriminant analysis of principal component (DAPC) without any *a priori* clustering condition was computed. The “DAPC” and the “find.clusters” functions within the adegenet R package v2.1.1 (https://cran.r-project.org/web/packages/adegenet/index.html) were used to compute the DAPC and determine the optimal cluster assignment.

Then, the same multivariate analysis was run on genera abundances by superimposing as *a priori* condition; the belonging of each sample according to metadata information was on maternal factors (BMI and gestational WG), delivery mode (VD and CS), feeding (BF and FF), weaning (≤4 and >4 months), and presence of older siblings (FB and nFB). Thus, in order to ascertain if DAPC classification was consistent with the original clusters and based on the discriminant functions, the “assignplot” function in the R “adegenet” package was used to calculate the proportions of successful reassignments.

## Results

The amplicon 16S rRNA sequencing analysis, performed on the 53 fecal samples of infants at 6 months of age (T2), determined a number of reads singularly assigned taxonomy that passed the quality control (QC) filter corresponding to 97,753.21 ± 22,044.56 (mean ± standard deviation) *per* sample. Of these, the 91.70 ± 2.13% (mean ± s.d.) was assigned at least to the genus level. To investigate factors influencing early microbiota colonization of infants during the first 6 months of life, samples were grouped based on: maternal factors [pre-pregnancy body mass index (BMI) and gestational weight gain (WG)], type of delivery, diet-related factors (the type of feeding and weaning), and presence/absence of older siblings in the household.

**Figure 1:**
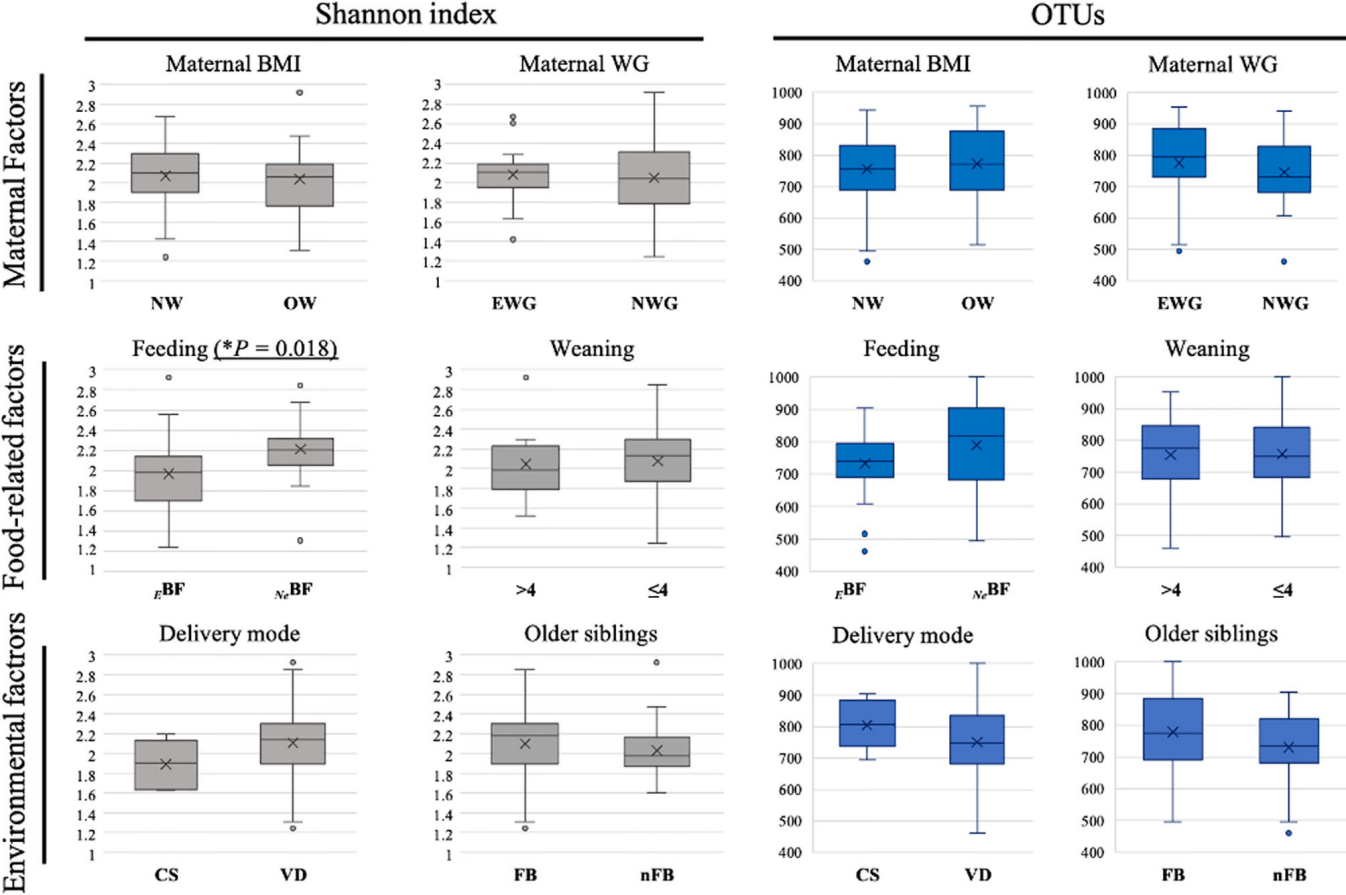
Box plots of the alpha diversity (Shannon index and number of operational taxonomic units (OTUs) identified) among T2 fecal samples (infants at 6 months of age) grouped according to different factors influencing early microbiota composition. Maternal factors: maternal pre-pregnancy body mass index (BMI) and gestational weight gain (WG). NW *[n.37]*: normal pre-pregnancy BMI (BMI < 25 kg/m^2^), OW *[n.12]*: excessive pre-pregnancy BMI (BMI ≥ 25 kg/m^2^); EWG *[n.20]*: excessive gestational WG, NWG *[n.29]*: optimal gestational WG). Food-related factors: Feeding (*_E_*BF *[n.31]*: exclusive breast-feeding, NeBF *[n.21]*: not exclusively breast fed, mixed fed or exclusively formula fed) and Weaning (≤4 *[n.41]*: before 4 months of age, >4 *[n.9]*: after 4 months of age). Environmental factors: Delivery mode (CS *[n.7]*: cesarean section, VD *[n.46]*: vaginal delivery) and presence of Older siblings (FB *[n.30]*: first-born, nFB *[n.22]*: not first-born). *P = p-value.

Alpha diversity, evaluated using the Shannon index and the number of operational taxonomic units (OTUs), was determined according to the different aforementioned factors (**Figure 1**). We observed that only *_E_*BF determined a lowering in Shannon index values (*P* = 0.018) when compared to not exclusively breastfed ones (*_Ne_*BF). Any other of the evaluated factors determined significant differences concerning alpha diversity.

### Factors Affecting Early Microbial Colonization of Infant Gut Microbiota

**Figure 2:**
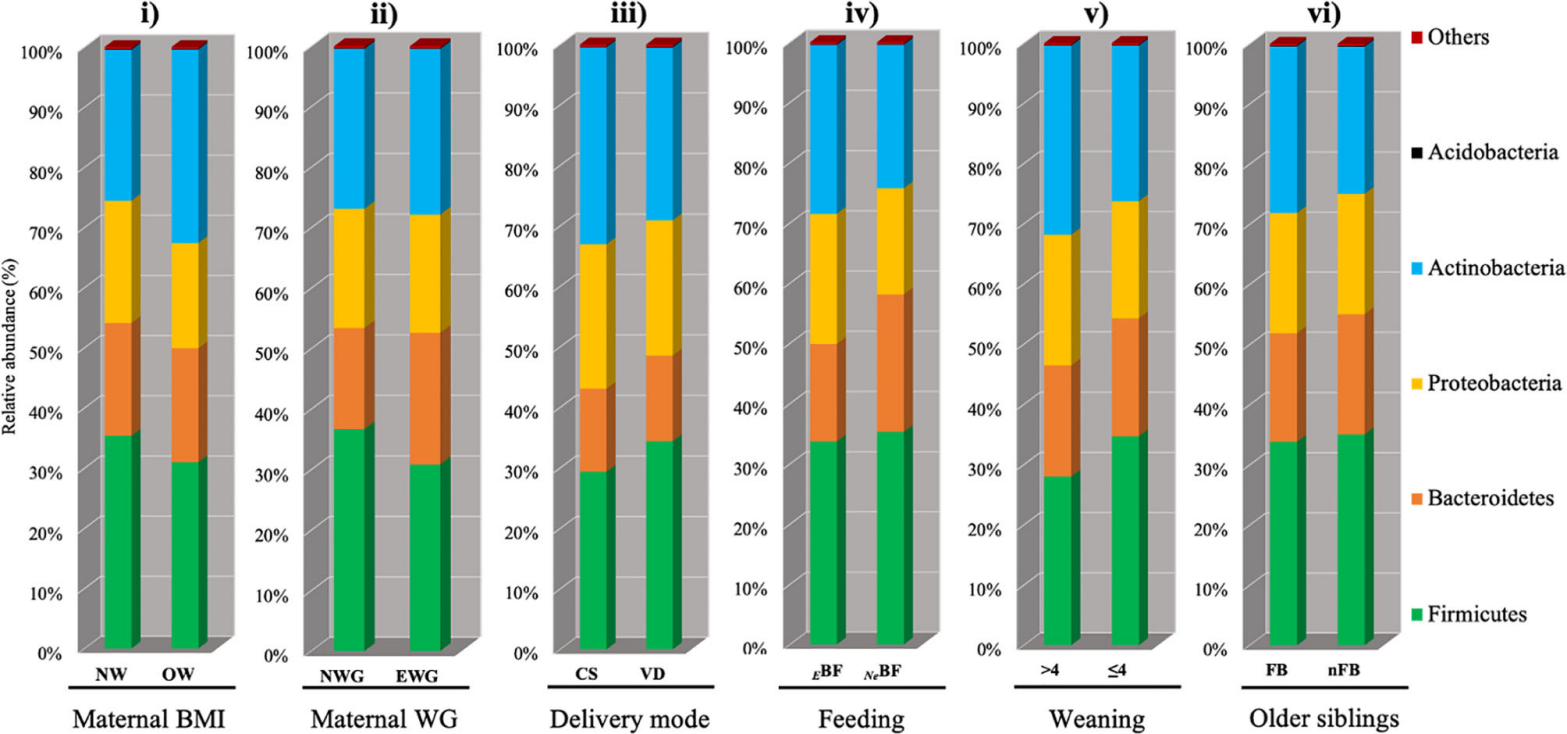
Influence of different factors on early microbiota composition at the phylum level (16S rRNA gene amplicon sequencing) in T2 fecal samples (infants at 6 months of age). Starting from the left: i) and ii) Maternal factors: maternal pre-pregnancy body mass index (BMI) and gestational weight gain (WG), respectively. NW *[n.37]*: normal pre-pregnancy BMI (BMI < 25 kg/m^2^), OW *[n.12]*: excessive pre-pregnancy BMI (BMI ≥ 25 kg/m^2^); EWG *[n.20]*: excessive gestational WG, NWG *[n.29]*: optimal gestational WG). iii) Delivery mode (CS *[n.7]*: caesarean section, VD *[n.46]*: vaginal delivery). iv) Feeding (*_E_*BF *[n.31]*: exclusive breast-feeding, NeBF *[n.21]*: not exclusively breast fed, mixed fed or exclusively formula fed). v) Weaning (≤4 *[n.41]*: before 4 months of age, >4 *[n.9*: after 4 months of age). vi) Presence of older siblings (FB *[n.30]*: first-born, nFB *[n.22]*: not first-born).

Starting from prenatal factors, maternal pre-pregnancy BMI within recommended values (BMI < 25 kg/m^2^) or higher (BMI ≥ 25 kg/m^2^) and gestational WG, optimal or excessive according to IOM reference values, were used to evaluate influence of maternal weight on gut microbiota composition in offspring till six months of age. At the phylum level, we did not find statistically significant differences concerning maternal features in both pre-pregnancy BMI and gestational WG (**Figures 2i, ii**). According to BMI and WG, any significant difference was found at the deeper taxonomic levels, specifically family and genus (data not shown).

Differently, evaluating infants’ microbiota composition at six months of age considering delivery mode (VD *vs* CS), an increased amount of Bacteroidetes in VD was detected (*P* = 0.021; **Table 1**, **Figure 2iii**). Despite this result at the phylum level, no other statistically significant difference at deeper taxonomic levels was detected.

**Table 1:** Statistically different phyla, families, and genera (16S rRNA gene amplicon sequencing) found in fecal samples of infants at 6 months of age (T2).

Taxon	metadata	feature	value	coef	Stderr	N	pval	qval	[factor] IQR (median)	[factor] IQR (median)
									**[CS]**	**[VD]**
Phylum	**Delivery**	Bacteroidetes	VD	0.85	0.36	53	0.021	0.209	0.38–0.87 (0.45)	0.44–37.1 (14.86)
									(**FB**)	(**nFB**)
Family	**Firstborn**	*Clostridiales* *Incertae Sedis XIII*	nFB	−0.12	0.03	53	0.001	0.108	0.01–0.01 (0.01)	0.00–0.01 (0.01)
	**Firstborn**	*Clostridiaceae*	nFB	−0.50	0.16	53	0.003	0.130	0.31–4.06 (0.81)	0.19–0.55 (0.23)
	**Firstborn**	*Peptostreptococcaceae*	nFB	−0.62	0.19	53	0.002	0.130	0.04–0.88 (0.32)	0.02–0.08 (0.04)
	**Firstborn**	*Planctomycetaceae*	nFB	0.10	0.03	53	0.003	0.130	ND*	0.00–0.01 (0.00)
									**[*_E_*BF]**	**[*_Ne_*BF]**
	**Feeding**	*Ruminococcaceae*	***_Ne_*BF**	0.63	0.15	53	<0.0001	0.011	0.12–0.22 (0.15)	0.19–2.19 (1.41)
Genus	**Delivery**	*Propionibacterium*	VD	−0.22	0.05	53	<0.001	0.031	**[CS]** 0.00–0.01 (0.00)	**[VD]** ND*
	**Delivery**	*Thiomicrospira*	VD	−0.15	0.04	53	<0.001	0.031	0.00–0.01 (0.01)	ND*
	**Delivery**	*Streptacidiphilus*	VD	−0.23	0.07	53	0.002	0.210	0.00–0.02 (0.01)	0.00–0.01 (0.00)
									**[*_E_*BF]**	**[*_Ne_*BF]**
	**Feeding**	*Flavonifractor*	***_Ne_*BF**	0.99	0.25	53	<0.001	0.112	0.00–0.01 (0.00)	0.01–1.25 (0.06)
	**Feeding**	*Erysipelotrichaceae* *Incertae Sedis*	***_Ne_*BF**	0.79	0.25	53	0.003	0.218	0.01–0.01 (0.01)	0.01-1.24 (0.09)
	**Feeding**	*Romboutsia*	***_Ne_*BF**	0.59	0.18	53	0.002	0.218	0.00–0.01 (0.00)	0.00–0.22 (0.02)
	**Feeding**	*Staphylococcus*	***_Ne_*BF**	−0.51	0.17	53	0.003	0.218	0.01–0.15 (0.02)	0.00–0.01 (0.01)
	**Feeding**	*Faecalicoccus*	***_Ne_*BF**	0.21	0.06	53	0.002	0.218	ND*	0.00–0.01 (0.00)
	**Feeding**	*Sulfurimonas*	***_Ne_*BF**	0.15	0.05	53	0.003	0.218	0.00–0.01 (0.00)	0.01–0.01 (0.01)
	**Feeding**	*Clostridium_IV*	***_Ne_*BF**	0.47	0.16	53	0.005	0.232	0.01–0.04 (0.01)	0.02–0.11 (0.06)
	**Feeding**	*Oribacterium*	***_Ne_*BF**	0.22	0.08	53	0.005	0.232	0.01–0.02 (0.01)	0.02–0.03 (0.02)

VD, vaginal delivery; CS, caesarean section; _E_BF, exclusive breastfed; _Ne_BF, combined or exclusive formula-feeding; FB, first-born; nFB, not first-born; IQR, interquartile range (25^th^–75^th^ percentile). *ND: not detected within all samples belonging to the considered group.

Evaluating type of feeding, specifically *_E_*BF or not *_Ne_*BF (mixed or exclusively formula-fed), no differences were found at the phylum level **Figure 2iv**. Interestingly, the family of *Ruminococcaceae* was strongly associated with the *_Ne_*BF group (*P* < 0.0001, qvalue = 0.011; **Table 1**). According to the family abundances, *Ruminococcaceae* subclusters were more abundant in FF, specifically *Flavonifractor* and the *Clostridium cluster IV* of Firmicutes. Also, other genera of Firmicutes mainly characterized the microbiota of FF infants, specifically *Faecalicoccus* (and other taxa of *Erysipelotrichaceae Incertae Sedis*), *Romboutsia*, and *Oribacterium* (*P* < 0.005). Contrarily, *Staphylococcus* was higher in *_E_*BF than in *_Ne_*BF (*P* = 0.003; **Table 1**).

The introduction of solid foods at least at 2 months of age [infants weaned before 4 months of age (≤4)] was not sufficient to determine significant shifts in the microbiota composition when compared to infants weaned after the 4^th^ month of age **Figure 2v**.

Among environmental factors, the presence of older siblings in the household did not determine differences at the phylum level **Figure 2vi**. The main feature of firstborn babies (FBs) was a higher abundance of *Clostridiaceae*, *Clostridiales Incertae Sedis XIII*, and *Peptostreptococcaceae* (*P*<0.003) than infants with older siblings (nFBs).

Thus, to evaluate the significant multivariable association among all infants, considering metadata (maternal BMI, gestational WG, type of delivery, feeding, weaning, and the presence of older siblings) as fixed effects in a regression model, we run MaAsLin2 software using family and genus relative abundances. Among all samples and relative metadata, *Ruminococcaceae* better discriminated _Ne_BF samples (*P* < 10^-3^; **Table S1**). However, this result was not confirmed for adjusted p-values (qval > 0.05). In the same line,* Flavonifractor* confirmed the previous discrimination in _Ne_BF samples (**Table S2**).

**Figure 3:**
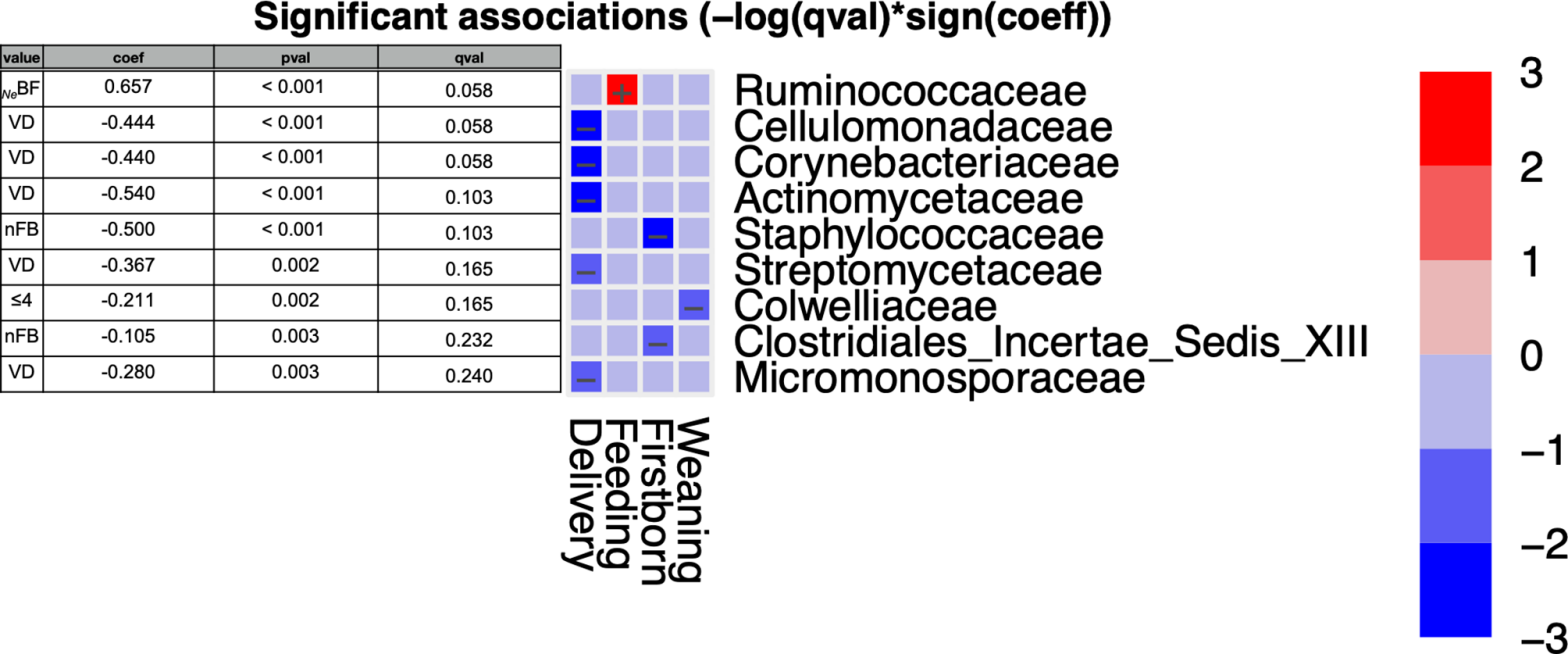
MaAsLin2 significant results and associations between postnatal factors (delivery, feeding, weaning, and presence of older siblings) and gut microbiota composition at the family level of infants at six months of age (T2). Based on normalized obtained significant results, the color scale-bar showed a positive relationship (red) and a negative one (blue) between taxa and factors, ranging from the highest positive normalization (+3) to the lowest one (−3).

As previously reported, prenatal factors (both maternal pre-pregnancy BMI and gestational WG) did not show any significant difference. For this reason, we developed a statistical analysis using as fixed metadata only postnatal factors (mode of delivery, type of feeding, weaning, and the presence of older siblings). Among families, it was confirmed that *Ruminococcaeae* positively correlated with *_Ne_*BF infants (**Figure 3**). Additionally, five families were negatively associated with vaginally delivered infants: *Cellulomonadaceae*, *Corynebacteriaceae*, *Actinomycetaceae*, *Streptomycetaceae*, and *Micromonosporaceae* (**Figure 3**). The presence of *Colwelliaceae* was negatively associated with infants weaned before 4 months of age. Meanwhile, *Staphylococcaceae* and *Clostridiales Incertae Sedis XIII* were negatively associated with nFB gut microbiota (**Figure 3**). However, all the aforementioned results did not confirm the significance for adjusted p-values (P < 0.05; qval > 0.05).

**Figure 4:**
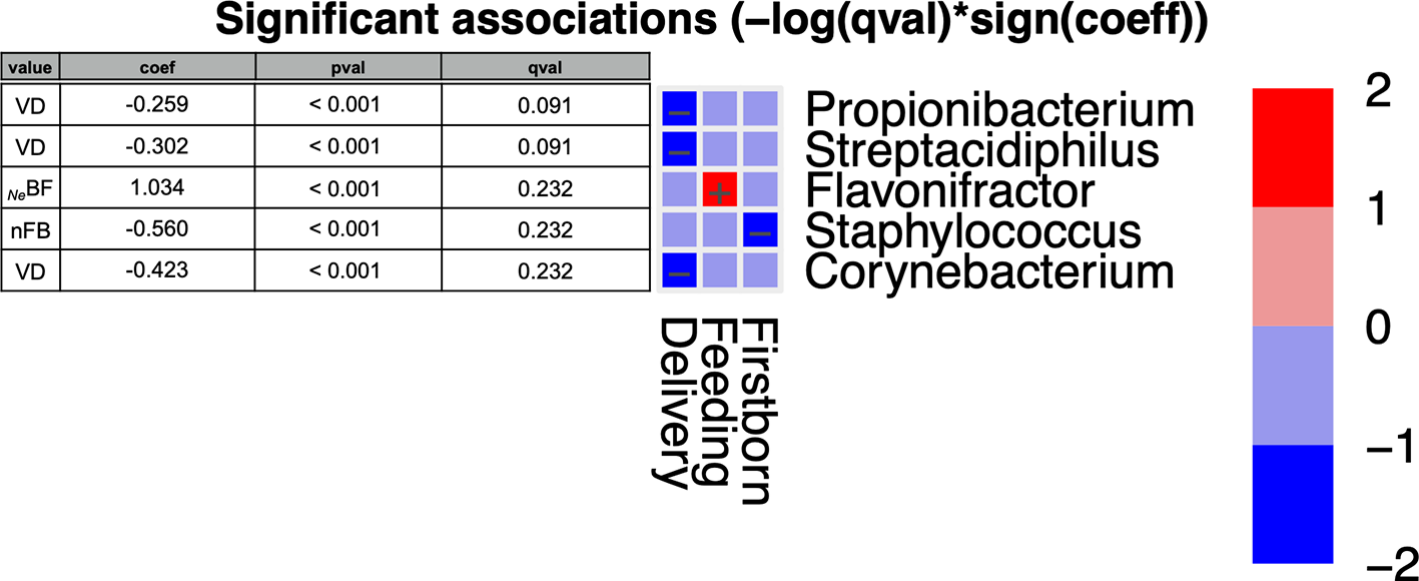
MaAsLin2 significant results and associations between postnatal factors (delivery, feeding, weaning, and presence of older siblings) and gut microbiota composition at the genus level of infants at six months of age (T2). Based on normalized obtained significant results, the color scale-bar showed a positive relationship (red) and a negative one (blue) between taxa and factors, ranging from the highest positive normalization (+2) to the lowest one (−2).

Among *_Ne_*BF (**Figure 4**), *Flavonifractor* exhibited a trend similar to *Ruminococcaceae* in *_Ne_*BF. Moreover, three genera were negatively associated with vaginally delivered infants (*Corynebacterium*, *Propionibacterium*, and *Streptacidiphilus*), while *Staphylococcus* was negatively associated with nFB microbiota. Otherwise, as reported in family results, also at the genus level the significance was not confirmed for adjusted p-values (P < 0.05; qval > 0.05).

### Multivariate Analyses

**Figure 5:**
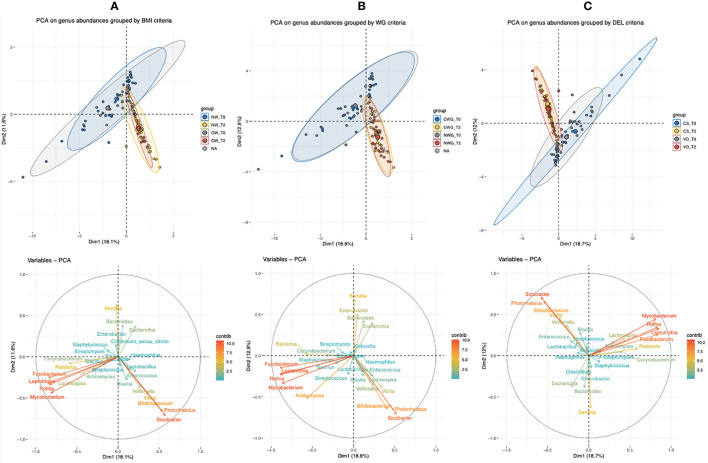
Principal component analysis (PCA) of bacterial genera with a median abundance >0.1% (16S rRNA gene amplicon sequencing) of infants’ T0 (meconium) and T2 (six months of age). Infants were grouped based on the relative metadata: **(A)** maternal pre-pregnancy BMI (NW, normal BMI, or OW, mothers with overweight/obesity), **(B)** maternal gestational weight gain (NWG, normal WG, or EWG, excessive WG), and **(C)** type of delivery (VD, vaginally delivered, or CS, by caesarean section).

To compare infant microbiota composition from birth to six months of age, we performed a multivariate analysis (PCA) between T2 and baseline T0 (*meconium*) samples. We analyzed maternal pre-pregnancy BMI (**Figure 5A**), maternal gestational WG (**Figure 5B**), and delivery type (**Figure 5C**), using as variables the genera with a median relative abundance greater than 0.1% at least in one of the sampled times (T0 or T2).

As shown by the PCA group ellipses, in all of the three considered conditions, time of sampling impacts deeper on sample stratification than on related metadata (BMI, WG, and delivery). Among the variables, *Fusobacterium*, *Leptotrichia*, *Mycobacterium*, *Serratia*, and *Rothia* had a deeper impact on T0-sample spatial distribution, whereas the genera *Bifidobacterium*, *Siccibacter*, *Photorabdus*, *Veillonella*, and *Vibrio* mainly characterized infants’ gut microbiota at T2.

Considering both T0 groups (NW and OW), the presence of four samples (three NW and one OW), that seem to be outliers, determined a not complete overlapping of both T0 clouds. On the other side, *Bifidobacterium*, *Siccibacter*, and *Photorabdus* relative abundances contributed to shifting a subset of NW samples at T2, therefore, determining elongation of the NW cloud (**Figure 5A**).

We also observed that at both times T0 and T2 the ellipses of NWG and EWG overlapped, and therefore, no PCA differences were associated with weight gain (**Figure 5B**).

Considering the type of delivery, CS and VD samples exhibited a more heterogeneous microbiota at T0, whereas at T2 the two delivery conditions allowed the construction of reduced and almost completely overlapped clouds (**Figure 5C**).

Therefore, a multivariate analysis was also run only on T2 fecal sample-set (infants at 6 months of age) performing a PCA and using bacterial genera (16S rRNA gene amplicon sequences) with a median value of relative abundance greater than 0.1% and therefore contributing to describe at least 50% of the whole population, resulting in 17 bacterial genera (**Figure S1b**). The two PCA principal components (Dim1 and Dim2; **Figure S1a**) described 52% of the total variance. The cos2 graduated scale values computed with R factoextra package described the quality of the sample. The best PCA sample quality is determined by high cos2 relative values. Observing the homogeneity of the sample in the PCA score plot where only a few linear distances marked some samples as outliers, it has been decided to perform another and more sensitive multivariate analysis. To understand how the aforementioned factors (maternal and diet-related ones, as well as environmental ones) affected infants’ early microbial colonization, the samples were clustered. By using the same filtered (median > 0.1%) set of genera, (whose vectors have been plotted in PCA biplot pf samples and variables, **Figure S1b**), discriminant analysis of the principal components (DAPC) was considered. Hence, a DAPC without superimposing any *a priori* condition was performed. As a result of the best fit cluster number identification, the “find.clusters” R function provided that four was the best-supported cluster number for the sample set. This information was used to run the DAPC. In the DAPC scatter plot (**Figure S2**) only cluster “4” was poorly populated (three samples), while the other three clusters (1, 2, and 3) included at least nine samples and were all placed into different quarters of the axes.

**Figure 6:**
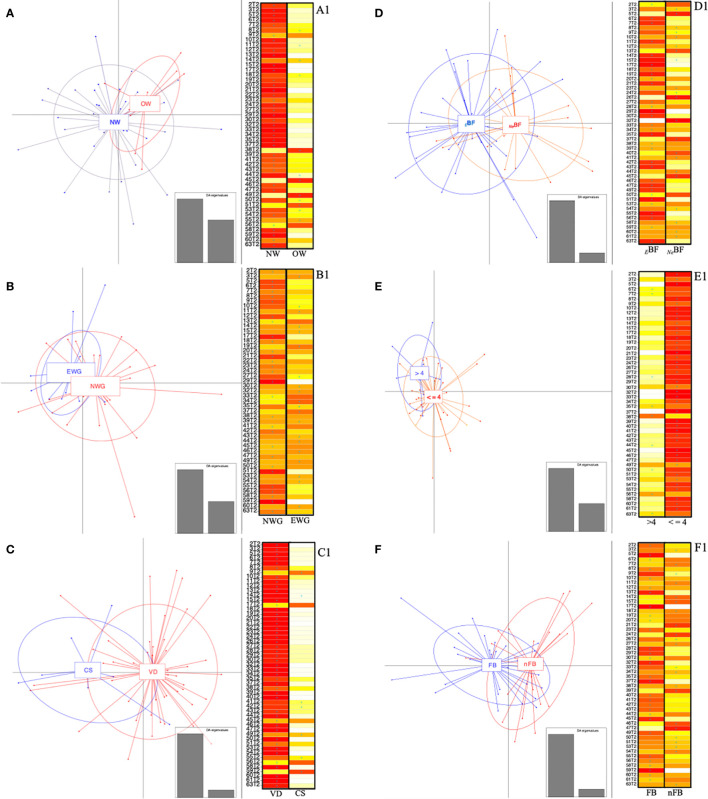
Discriminant analysis of principal components (DAPC) and relative score-plots. **(A–F)** report the DAPC score plots based on the relative abundances (16S rRNA gene amplicon) of genera with median values >0.1% found in infants at 6 months. Panel: **(A)** NW, normal BMI (<25 kg/m^2^); OW, women overweight/obesity (BMI ≥ 25 kg/m^2^); **(B)** NWG, optimal weight gain; EWG, excessive weight gain; **(C)** VD, vaginally delivered; CS, delivered by cesarean section; **(D)** BF, exclusive breast-feeding; FF, combined or exclusive formula-feeding; **(E)** ≤4, infants weaned before 4 months of age; >4, infants weaned after 4 months of age. Panel **(F)** FB, first-born; nFB, not first-born. **(A1–F1)** respectively indicate whether the infants (rows) could be assigned based on discriminant functions by K-means analysis. The cell colors represent the membership probabilities (K-means analysis) to belong to each cluster (red = 1, orange = 0.75, yellow = 0.25, white = 0) and blue crosses indicate the originally belonging cluster.

In a second approach, each sample was assigned the membership group, as *a priori* condition, and the same was repeated for all metadata. The tested conditions (both maternal factors, type of delivery, dietary features, and presence of older sibling) resulted in the configuration of overlapping clusters (**Figures 6A–F**). Looking at the DAPC score plot concerning maternal BMI (**Figure 6A**), it was possible to observe that the samples belonging to NW were more divergent among themselves, ensuing an enlargement of the ellipse and, therefore, determining a partial overlapping on the OW-ellipse. Otherwise, none of the NW samples was assigned to the OW group; specifically 66% of OW samples should be included in the NW group (**Figure 6A1**).

Similarly, when evaluating the WG variable, we found that the EWG ellipse was included in the NWG one (**Figure 6B**). For 40% of the EWG samples, the *a priori* assignment was not verified, thus implying a better fit to the NWG group. On the opposite, 17% of EWG was not verified as part of the *a priori* assigned group (**Figure 6B1**).

The analysis based on the delivery mode reported a divergence of the ellipses of VD and CS (**Figure 6C**), displaying the relative centers in a different quarter of the DAPC system. Evaluating the “assign plot” scores in five out of seven CS samples, we observed a better fit to the VD group, while DAPC K-means analyses membership was not confirmed only in 6.5% of VD samples (**Figure 6C1**).

Concerning the feeding, the percentages of overlapping were only 19% in *_E_*BF, whereas 50% of *_Ne_*BF looked like they were fitted to the *_E_*BF group (**Figure 6D1**); meanwhile, seven out of nine infants weaned after 4 months of life might be included in the ≤4 group and no one of this second group (≤4) showed a controversial membership (**Figure 6E1**).

We also analyzed the presence/absence of older siblings considering the “hygiene hypothesis” (Strachan, 1989). For these superimposed groups, the ellipsoid centers of the two clusters (FB and nFB) were placed into different plot quarters (**Figure 6F**). Also, by inspecting the relative “assign plot” sample membership probabilities, we observed the *a priori* clustering condition was not verified in 23 and 32% of FB and nFB, respectively (**Figure 6F1**).

## Discussion

A great relevance is widely assigned to the gastrointestinal (GI) microbiome composition’s impact on health (Young, 2017), and it is well known that early microbial colonization could significantly contribute to long-term healthy and unhealthy consequences during lifespan (Tamburini et al., 2016). Infant GI microbiota colonization is crucial for healthy growth and is primarily involved in gut maturation and immune system development; indeed, altered colonization has been associated with a high risk of disease outbreaks later in life (Stiemsma and Michels, 2018). In this line, the present longitudinal and observational study was aimed at evaluating the influence of maternal factors (pre-pregnancy BMI and gestational WG), perinatal factors such as type of delivery, infant’s diet (the type of feeding and weaning timing), and the presence/absence of older siblings in the household, on GI microbiota composition of babies after six months of life (T2).

Concerning the prenatal factors, such as maternal pre-pregnancy BMI and gestational WG, evidence showed that children born from mothers with overweight/obesity have an increased risk to develop obesity during their life (Ajslev et al., 2011) even if the factors behind this relationship are not fully understood. In our study, sampling infants at 6 months of age, we did not find differences in alpha diversity according to maternal factors (pre-pregnancy BMI and gestational WG). Additionally, we detected only partial and not significant differences in Firmicutes and Bacteroidetes abundances in offspring in line with previous studies, which reported no difference in F/B ratio in obese *versus* lean humans and rodents (Duncan et al., 2008; Jumpertz et al., 2011; Zhang et al., 2012). Other evidence observed Firmicutes overabundant in obese mice (Ley et al., 2005) and that microbiota of obese mice and lean littermates encoded different metabolic pathways (Turnbaugh et al., 2006). On the contrary, Collado and coworkers observed high abundances of Bacteroidetes subtaxa in overweight mothers and relative offspring (Collado et al., 2008; Collado et al., 2010); therefore, we emphasize that the reason stands behind the deeper taxonomic levels (*i.e.*, genus and species), where taxa and relative genes directly contribute to obesity onset.

In line with Collado et al., a significant overabundance of *Parabacteroides* and *Bacteroides* in fecal samples of children born from mothers with an excessive BMI has been reported also in another study (Cerdò et al., 2018). Both these Bacteroidetes genera are microbes that may frequently be vertically transmitted from mothers to offspring (Nayfach et al., 2016; Li et al., 2020), and for this reason, the presence of these taxa in the maternal GI microbiota may affect their presence also in newborns (Collado et al., 2010). Considering vertical transmission of microbes, we found that Bacteroidetes mainly characterized the gut microbiota of vaginally delivered infants. As Jakobsson et al. reported (Jakobsson et al., 2014), vaginally delivered infants were more characterized by high abundance of Bacteroidetes and relative subtaxa (*e.g.*, *Bacteroides*) than CS delivered ones. Interactions between Bacteroidetes and host seem to be crucial at the beginning of life due to the fact that *Bacteroides* may be able to correct the presence of the underdeveloped immune system in germ-free mice (Ivanov et al., 2008).

Overall, the main key factor able to improve and boost up the immune system certainly is exclusive breastfeeding. The World Health Organization (WHO) recommends that infants should be exclusively breastfed during the first 6 months of life (Butte et al., 2002) for the beneficial effects of breastfeeding on the immune system programming (Laouar, 2020). In the present study, *_Ne_*BF samples reported higher values of Shannon index compared to *_E_*BF ones. In adults’ microbiota, high values of alpha diversity are positively related to ecosystem resistance, resilience, and health (Shade et al., 2012); however, in the early phases of life, it has been previously observed that formula-feeding determines high values of alpha diversity indices (Bezirtzoglou et al., 2011; Fan et al., 2013). Breastfed infants, indeed, are principally characterized by *Bifidobacterium* species (Gueimonde et al., 2007); therefore, their presence markedly affects, reducing, alpha diversity (Bezirtzoglou et al., 2011). This class of probiotics is markedly able to metabolize HMOs or HMO constituents (Duranti et al., 2019), prevailing on other taxa, and this is the power of human milk feeding. In the present study, we did not observe a high abundance of *Bifidobacteriaceae* (or relative subtaxa) in *_E_*BF infants, probably due to the fact that our *_Ne_*BF group included also infants fed with combine feeding (mixed feeding, *i.e.*, formula and breast milk). This condition could have reduced the clustering of our samples based on the type of feeding. However, despite this possible overlapping, we observed that our *_Ne_*BF group was mainly characterized by high abundances of *Ruminococcaceae*, in particular *Flavonifractor*, and taxa assigned to the *Clostridium cluster IV*. This is in line with previous studies (Fan et al., 2013; Tannock et al., 2013; Sagheddu et al., 2016; Durrani et al., 2020), in which *Ruminococcacae* or *Lachnospiraceae* has been reported to be higher in the absence of competition with *Bifidobacteriaceae*, replacing them as butyrogenic bacteria in the gut environment of *_Ne_*BF babies (Bui et al., 2020; Vacca et al., 2020). At the genus level, the high abundances of *Ruminococcaceae* determined a positive association between *_Ne_*BF and *Flavonifractor*. The presence of *Flavonifractor* in *_Ne_*BF infants has been previously found in different manuscripts (Zheng et al., 2016; Borewicz et al., 2019). This genus has been associated with a high level of circulating cytokines (Huang et al., 2019) and fecal microbiota of infants with food allergies (Ling et al., 2014). Recently, Bui et al. described an enrichment of the microbial pathways able to degrade *Nϵ*-fructosyllysine in stools of formula-fed infants, whereas fecal microbiota of exclusively breastfed was not able to grow on *Nϵ*-fructosyllysine medium (Bui et al., 2020). The authors also observed that in the gut microbiota of *_E_*BF infants there was a lack of *Intestinimonas*–*Flavonifractor*–*Pseudoflavonifractor* group, a clade that mainly characterized stools of formula-fed infants. The transfer of the *Nϵ*-fructosyllysine/lysine pathway genes seems to be vertical, from mothers to offspring (Yassour et al., 2016), but the selective outgrowth of *Nϵ*-fructosyllysine/lysine-fermenting microorganisms predominated in formula-fed infants, suggesting that the type of milk might influences their outgrowth. We also found a positive association between *_Ne_*BF and *Erysipelotrichaceae Incertae Sedis* (**Table 1**). In previous studies, also this taxon has been reported mainly in formula-fed gut microbiota (Wang et al., 2015; Borewicz et al., 2019) probably acting as *Ruminococcaceae* and *Lachnospiraceae* in the butyrate metabolism (Vital et al., 2017). Similar to *Flavonifractor*, Erysipelotrichaceae *Incertae Sedis* is likely linked to adverse outcomes concerning asthma and allergy later development (Dzidic et al., 2017).

Concerning weaning, previous research studies have shown that timing of solid food introduction likely plays a relevant role in the development of childhood overweight and obesity, with an increased risk when weaning process starts before 4 months of age (Wang et al., 2016; Differding et al., 2020). However, the aforementioned differences have been observed in babies aged at least 2 years. In our study on younger ones, we did not find any statistically significant difference resulting from an early introduction of solid foods. That result could undoubtedly emerge from the different feeding types (exclusive breast-, mixed-, or exclusive formula feeding) in the first six months of life, determining overlap in the microbiota composition. However, in our opinion, early weaning does not define any advantage in terms of GI microbiota maturation at six months of age. Therefore, considering the literature cited above, breastfeeding remains the gold standard for optimal nutrition in the first six months of age and microbiota shaping interests.

As regards early microbiota colonization, other research studies have shown some differences according to the presence/absence of older siblings in the household. Starting from the “hygiene hypothesis” (Strachan, 1989), different hypothesis focused on the impact of exposure to infections during the first years on aberrant immune responses later in life. Strachan observed that the presence of older siblings in the household decreased the risk to develop allergies in infants (Strachan, 2000). According to the results so far collected, in our study, babies with an older sibling at six months of age displayed lower amounts (*P*<0.05) of *Clostridiaceae*, *Peptostreptococcaceae*, *Clostridiales Incertae Sedis XIII*, clusters that typically colonize the gut lumen in the first days after birth (Penders et al., 2014; Milani et al., 2017). Other evidence reported an early maturation of the microbial colonization mediated by the “adult”-associated genus *Faecalibacterium* (Laursen et al., 2017). Therefore, we suggest that the presence of older siblings in the household contributes to expose infants to “other and new” environmental bacterial patterns and indirectly reduces the abundances of the neonatal microbial colonizer. Analyzing all postnatal factors and their effects on the gut microbial composition, both *Staphylococcaceae* and *Staphylococcus* were reported to have a negative and significant relationship with nFB (**Figures 3** and **4**). *Staphylococcus* is an early gut colonizer in neonates and in particular in BF infants (Balmer and Wharton, 1989); thus, the presence of older siblings seems to influence an early shift towards an “adult” microbiota profile in nFB compared to FB.

Taking into account the low power of the considered variables in clustering our samples when T0 and T2 were compared (**Figure 5**) to discern if some factors determined a clear microbial clustering, we also run a multivariate analysis only on T2 samples. According to maternal factors, we observed that both BMI and WG reported the greatest overlapping of the relative ellipses (**Figures 6A, B** **)**. Based on DAPC results, infants lost or reduced the significant relevance of prenatal features probably because of the postnatal events that occurred in the last 6 months of life. Also, the type of delivery did not show great strength in clustering our samples despite interesting results linked to over-presence of Bacteroidetes in VD were found. For those reasons the programmed T3 sampling of the A.MA.MI project (12 months of life of infants) will be essential to increase evidence of long-term effects linked to this finding.

As expected, the main finding obtained from the sampling of the gut microbiota of six-month-old babies is related to dietary features. One of the limits of our study is that the *_Ne_*BF group includes both exclusive and combined formula-fed infants; therefore we were not able to observe how exclusive breastfeeding could be able to improve the gut environment harboring or could suppress specific bacterial patterns; we could only assume this effect by looking at alpha diversity findings. Indeed, it is clear how feeding has great relevance in this transitional time point, highlighting these primary results. Although *Ruminococcaceae* are often associated with beneficial outcomes (Liu et al., 2020) based on their roles in butyrate metabolism in adults (Biddle et al., 2013), their presence in our cohort was not necessarily beneficial. Thus, the different and widely reported involvement of *Bifidobacteriaceae* in gut health remains a key factor to be considered in formula feeding.

Further, considering that we have studied infants only until six months of age, we cannot give evidence about a positive or negative contribution of early weaning in the infant’s gut microbiota.

Lastly, also DAPC performance based on the presence of older siblings showed interesting findings. Although no apparent clustering was found, DAPC determined a cluster separation of two groups (FB and nFB) into different system planes and associated admissible overlapping percentages with parents’ care. Thus, it is easily presumed that, although parents of firstborns could be more apprehensive, the attention and care provided are strictly correlated to the family’s cultural level and life experience. Moreover, the indirect contribution deriving from older siblings is not negligible under the point of view of the household “contamination” with other microbes.

Our study has some important strengths. First of all, our research was not based only on single-factor analysis able to shape early microbial composition. We adopted a multifactorial approach, taking into account the main prenatal and postnatal factors, providing a more extensive framework on this critical window of plasticity. Data analysis and result interpretation required multidisciplinary expertise in microbiology, clinical nutrition, pediatrics and biostatistics science, with a well-integrated approach. Nonetheless, some limitations need to be acknowledged. Only infant GI microbiota, and not maternal one, was analyzed. This determined a partial loss in fully interpreting the results, particularly those linked to maternal pre-pregnancy BMI, gestational WG, and the mode of delivery. Additionally, we did not investigate maternal antibiotic therapy during lactation, which might have influenced infant GI microbiota colonization. Lastly, the sample size was small and did not allow us to create homogenous groups for all the considered variables.

## Conclusion

The first year of life is crucial for healthy growth; several factors affect gut microbiota development in newborns in this critical time window. Prenatal factors and postnatal ones directly contribute to infants’ gut microbiota maturation determining possible disease outbreaks later in life. The type of milk feeding undoubtedly has a pivotal role in our population, preserving different microbiota composition after at least two months from solid food introduction. Although we found some differences in grouping samples considering maternal factors, diet-related ones, and presence/absence of older siblings, these did not help to identify specific actors to determine an absolute clustering of the belonging samples. Undoubtedly, exclusive breastfeeding preserved the gut microbiota composition mainly characterized by bifidobacteria, a condition that a formula feeding, both exclusive and combined, did not harbor. The risks associated with overgrowth of specific and not beneficial taxa at six months of life could indirectly contribute to future disease onset, therefore determining adverse long-term effects.

Our findings contribute to add evidence of the complex multifactorial interaction of different maternal and neonatal factors on GI microbiota composition at this age. Based on these considerations, the programmed “at one year”-sampling becomes essential to bring evidence on how the different prenatal and postnatal factors drive the intestinal microbiota.

## Data Availability Statement

The data are available in the NCBI database: http://www.ncbi.nlm.nih.gov/bioproject/675753; https://www.ncbi.nlm.nih.gov/sra/PRJNA675753, BioProject ID: PRJNA675753.

## Ethics Statement

The studies involving human participants were reviewed and approved by the Human Ethics Committee (EC) of Fondazione IRCCS Policlinico S. Matteo of Pavia. Written informed consent to participate in this study was provided by the participants’ legal guardian/next of kin.

## Author Contributions

BR and MV equally contributed to the conception and design of the study, results’ interpretation, and drafted the manuscript. DP and RD contributed to the conception, design of the study, results’ interpretation, and revised the manuscript. MC and ML contributed to results’ interpretation and revised the manuscript. RC, EC, and FG, contributed to the conception and design of the study and revised the manuscript. MD, MV, and FC conducted the statistical analysis, interpreted results, and revised the manuscript. HC drafted, revised, and approved the final version of the manuscript.

All authors have read and approved this version of the manuscript and declare that the content has not been published elsewhere. All authors contributed to the article and approved the submitted version.

## Conflict of Interest

The authors declare that the research was conducted in the absence of any commercial or financial relationships that could be construed as a potential conflict of interest.
