# Supplementary material for: Early Life Microbiota Colonization at Six Months of Age: A Transitional Time Point
Source: Front Cell Infect Microbiol. 2021 Mar 26;11:590202. doi: 10.3389/fcimb.2021.590202 (PMC8032992; doi:10.3389/fcimb.2021.590202)
Supplement: Supplementary file 1 [file DataSheet_1.pdf]

Filename: hellas1.cD1000

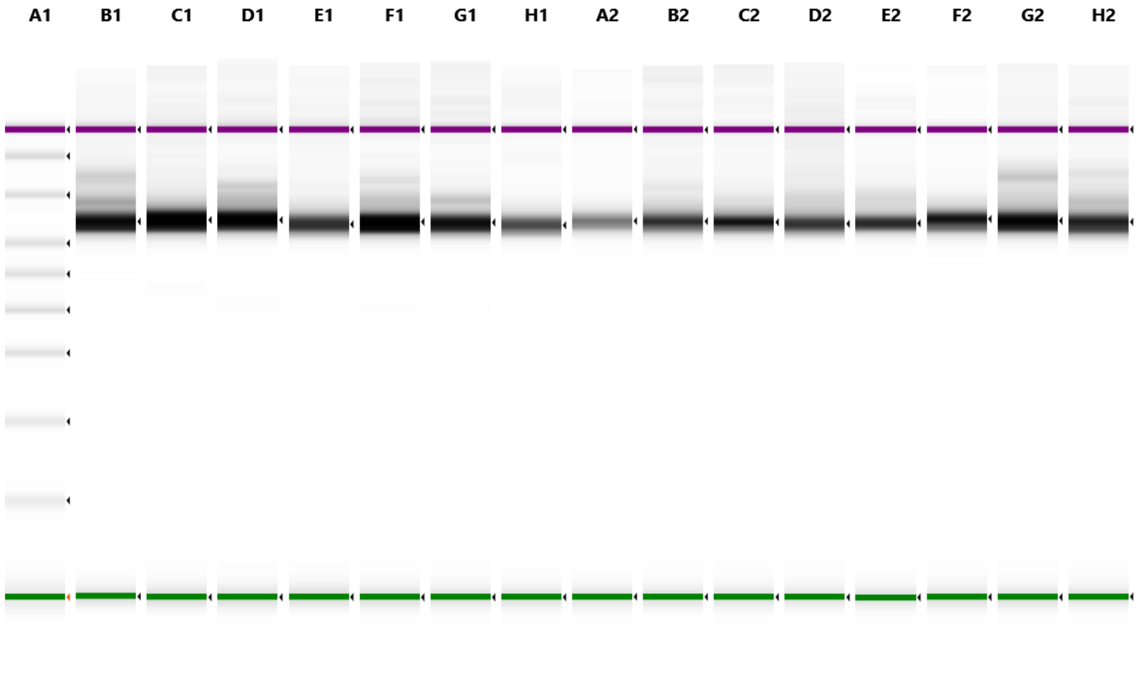

Default image (Contrast 100%)

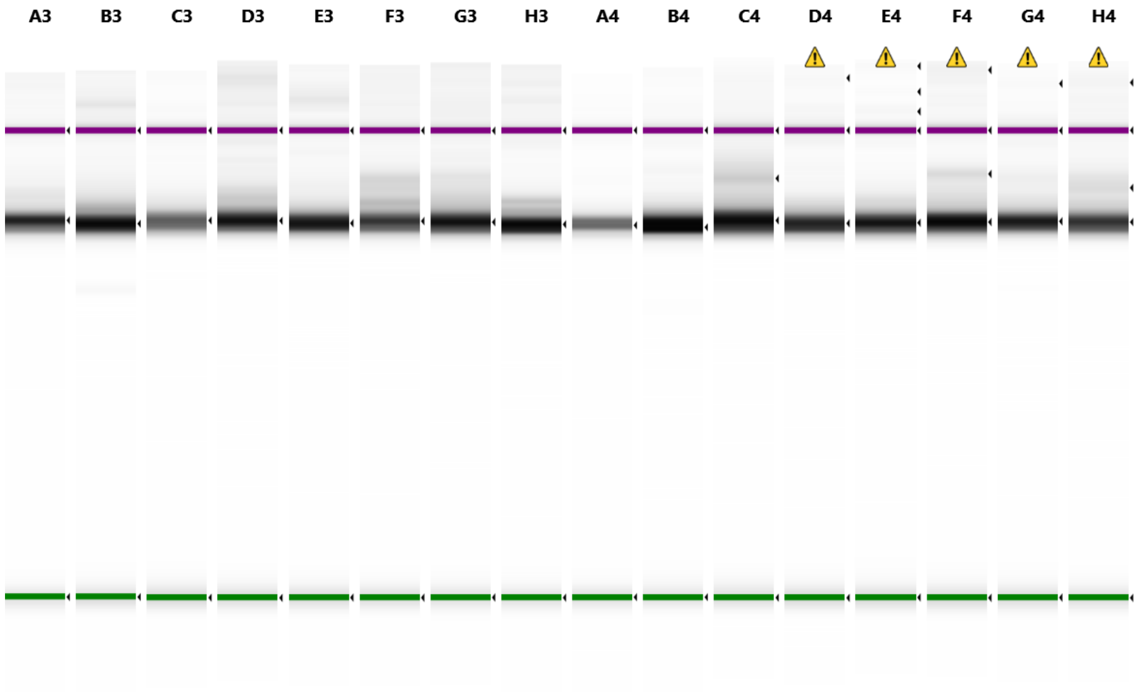

Default image (Contrast 100%)

© Agilent Technologies, Inc. 2019

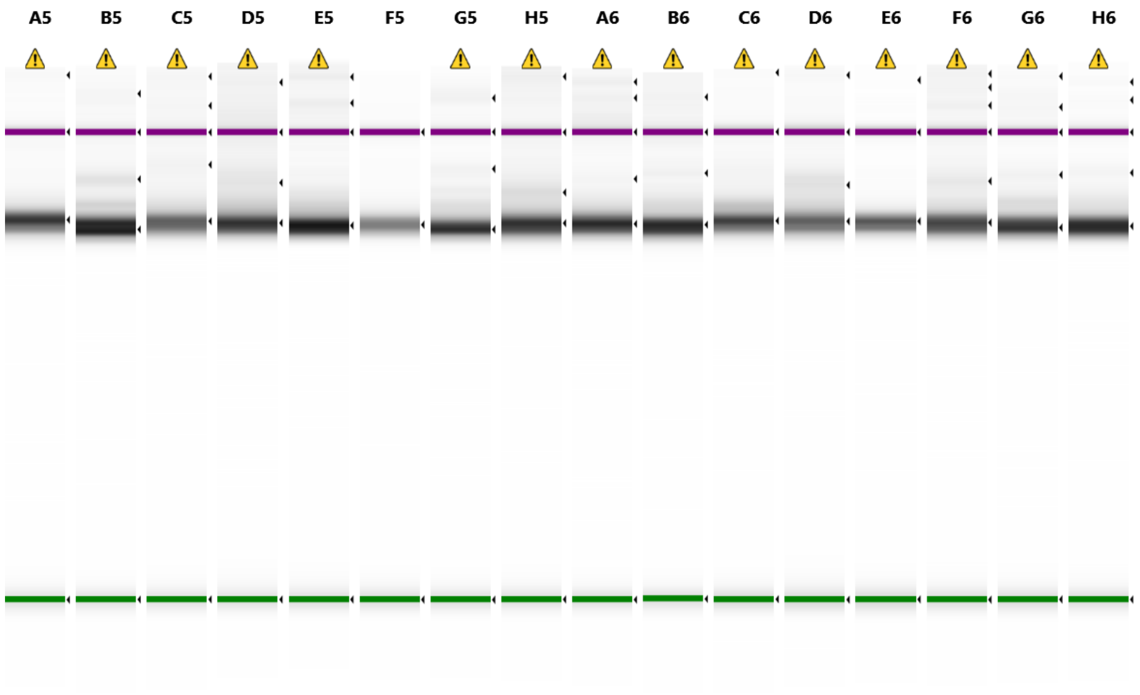

Default image (Contrast 100%)

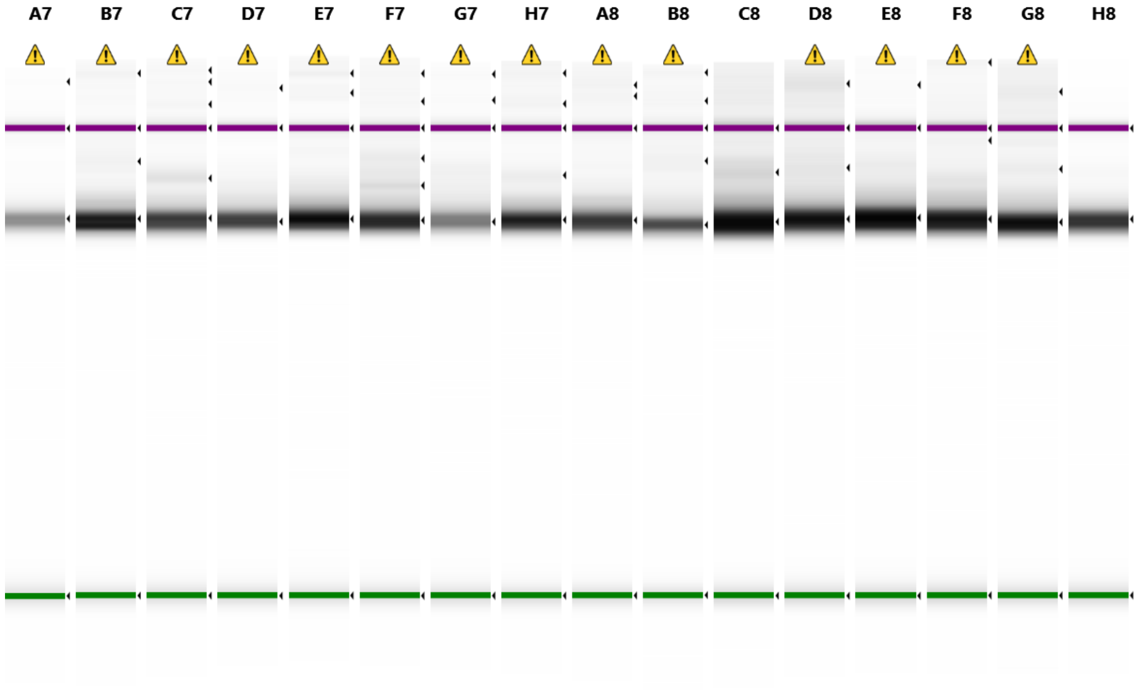

Default image (Contrast 100%)

© Agilent Technologies, Inc. 2019

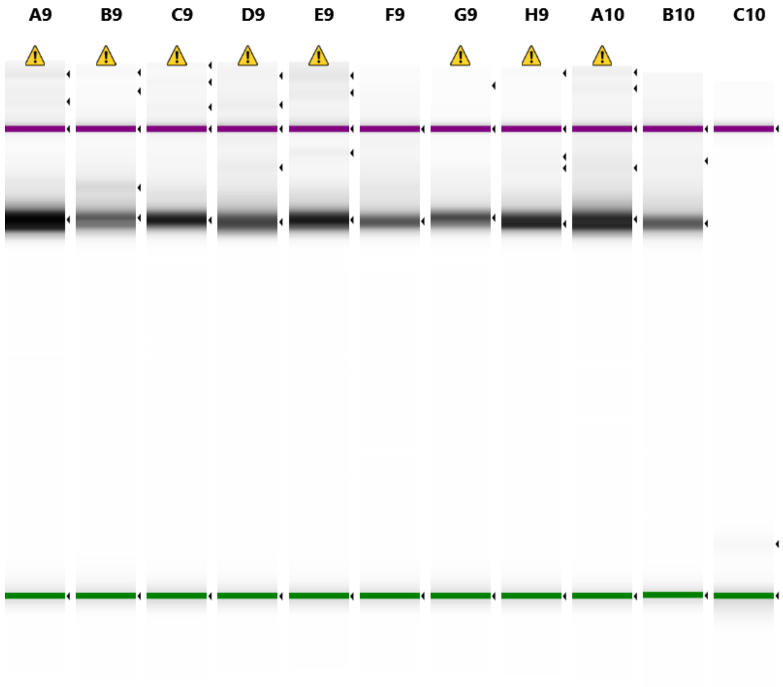

Default image (Contrast 100%)

© Agilent Technologies, Inc. 2019

Sample Info

Well	Conc. [ng/µl]	Sample Description	Alert	Observations
A1	20.3	Electronic Ladder		Ladder
B1	70.5	#37 T2		
C1	109	#27 T2		
D1	90.1	#44 T2		
E1	45.4	#9 T2		
F1	82.1	#10 T2		
G1	70.9	#8 T2		
H1	41.1	#17 T2		
A2	26.5	#35 T2		
B2	45.5	#15 T2		
C2	59.8	#6 T2		
D2	43.6	#13 T2		
E2	48.1	#32 T2		
F2	68.3	#14 T2		
G2	86.8	#60 T2		
H2	60.3	#23 T2		
A3	51.8	#12 T2		
B3	65.3	#34 T2		
C3	35.4	#30 T2		
D3	62.4	#21 T2		
E3	60.9	#28 T2		
F3	46.0	#50 T2		
G3	63.7	#40 T2		
H3	76.0	#55 T2		
A4	31.7	#41 T2		
B4	93.5	#46 T2		
C4	77.1	#2 T2		
D4	47.8	#49 T2	⚠	Peak out of Sizing Range
E4	61.2	#29 T2	⚠	Peak out of Sizing Range
F4	74.5	#43 T3	⚠	Peak out of Sizing Range
G4	55.5	#58 T3	⚠	Peak out of Sizing Range
H4	60.8	#33 T3	⚠	Peak out of Sizing Range
A5	51.5	#22 T3	⚠	Peak out of Sizing Range
B5	75.0	#37 T3	⚠	Peak out of Sizing Range
C5	43.5	#24 T3	⚠	Peak out of Sizing Range
D5	55.7	#15 T3	⚠	Peak out of Sizing Range
E5	70.3	#55 T3	⚠	Peak out of Sizing Range
F5	25.7	#34 T3		
G5	52.5	#26 T3	⚠	Peak out of Sizing Range
H5	59.8	#11 T3	⚠	Peak out of Sizing Range
A6	45.2	#19 T3	⚠	Peak out of Sizing Range
B6	49.3	#42 T3	⚠	Peak out of Sizing Range
C6	45.2	#5 T3	⚠	Peak out of Sizing Range
D6	40.0	#54 T3	⚠	Peak out of Sizing Range
E6	32.3	#50 T3	⚠	Peak out of Sizing Range
F6	49.7	#53 T3	⚠	Peak out of Sizing Range
G6	51.6	#18 T3	⚠	Peak out of Sizing Range
H6	59.1	#17 T3	⚠	Peak out of Sizing Range
A7	23.8	#13 T3	⚠	Peak out of Sizing Range
B7	47.6	#57 T3	⚠	Peak out of Sizing Range
C7	59.1	#10 T3	⚠	Peak out of Sizing Range
D7	53.0	#21 T3	⚠	Peak out of Sizing Range
E7	83.4	#46 T3	⚠	Peak out of Sizing Range
F7	73.4	#47 T3	⚠	Peak out of Sizing Range
G7	32.2	#7 T3	⚠	Peak out of Sizing Range
H7	67.5	#9 T3	⚠	Peak out of Sizing Range
A8	60.9	#14 T3	⚠	Peak out of Sizing Range
B8	42.5	#38 T3	⚠	Peak out of Sizing Range
C8	83.6	#23 T3		
D8	73.9	#2 T3	⚠	Peak out of Sizing Range
E8	85.7	#27 T3	⚠	Peak out of Sizing Range
F8	72.6	#61 T3	⚠	Peak out of Sizing Range
G8	76.3	#45 T3	⚠	Peak out of Sizing Range
H8	56.4	#20 T3		
A9	86.2	#39 T3	⚠	Peak out of Sizing Range
B9	44.9	#44 T3	⚠	Peak out of Sizing Range
C9	48.8	#29 T3	⚠	Peak out of Sizing Range
D9	47.3	#60 T3	⚠	Peak out of Sizing Range
E9	58.7	#6 T3	⚠	Peak out of Sizing Range
F9	31.0	#8 T3		
G9	37.4	#41 T3	⚠	Peak out of Sizing Range
H9	60.2	#63 T3	⚠	Peak out of Sizing Range

© Agilent Technologies, Inc. 2019

A10	74.3	#56 T3	⚠	Peak out of Sizing Range
B10	31.0	#35 T3		
C10	1.25	CTRL- H2O		

© Agilent Technologies, Inc. 2019

A1: Electronic Ladder

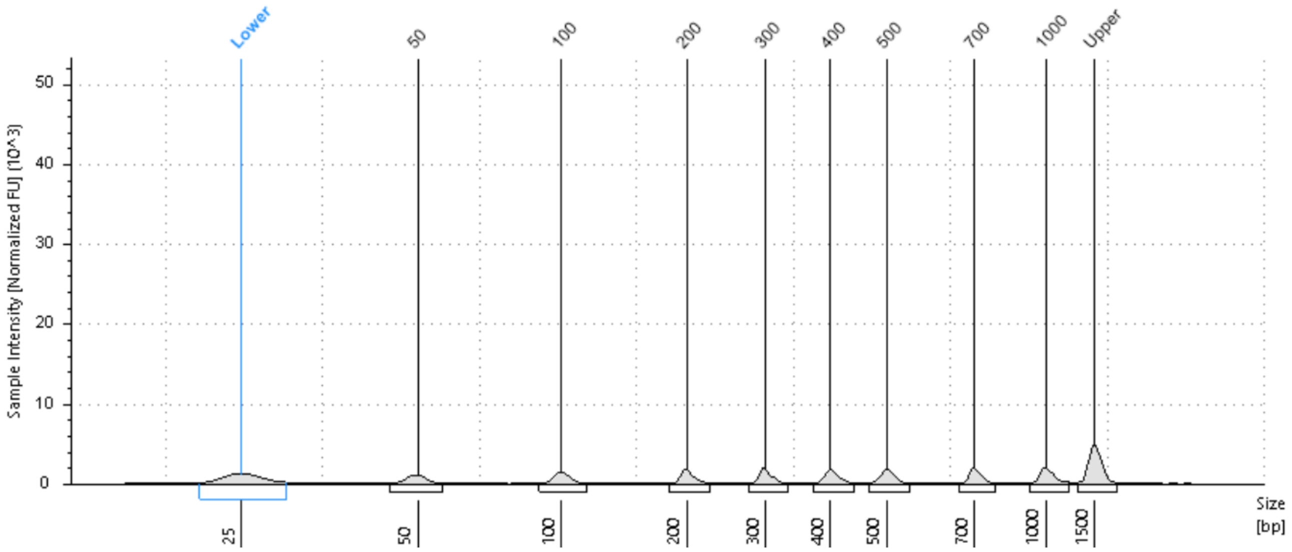

Sample Table

Well	Conc. [ng/µl]	Sample Description	Alert	Observations
A1	20.3	Electronic Ladder		Ladder

Peak Table

Size [bp]	Calibrated Conc. [ng/µl]	Assigned Conc. [ng/µl]	Peak Molarity [nmol/l]	% Integrated Area	Peak Comment	Observations
25	5.22	-	321	-		Lower Marker
50	2.25	-	69.3	11.11		
100	2.37	-	36.5	11.71		
200	2.47	-	19.0	12.20		
300	2.55	-	13.1	12.56		
400	2.57	-	9.87	12.66		
500	2.71	-	8.33	13.36		
700	2.46	-	5.41	12.15		
1000	2.89	-	4.44	14.25		
1500	6.50	6.50	6.67	-		Upper Marker

© Agilent Technologies, Inc. 2019

B1: #37 T2

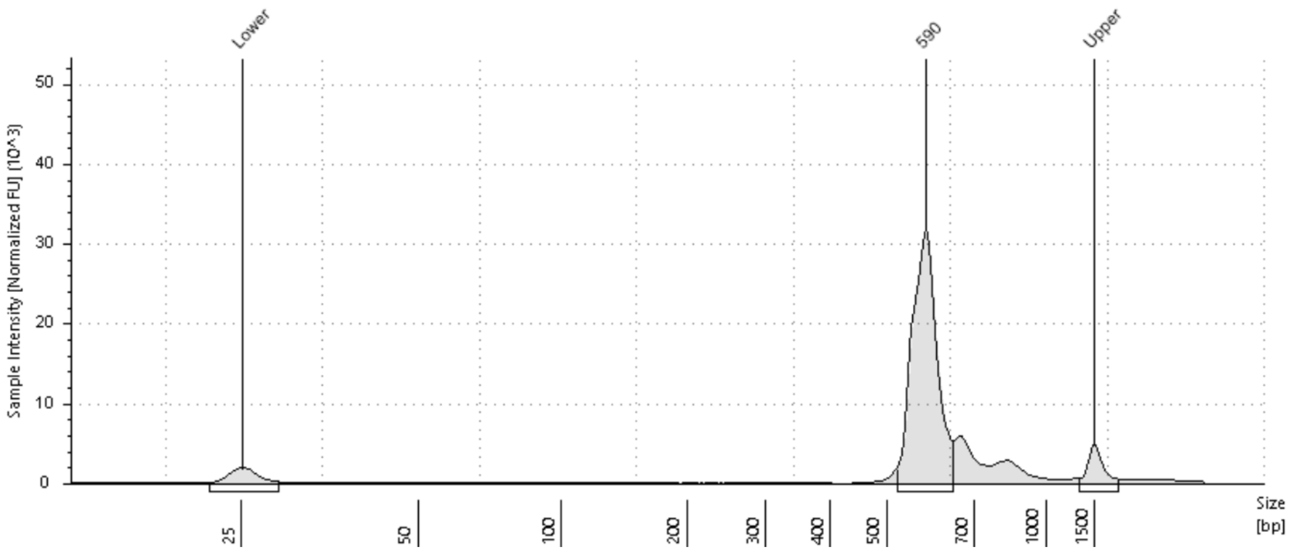

Sample Table

Well	Conc. [ng/µl]	Sample Description	Alert	Observations
B1	70.5	#37 T2		

Peak Table

Size [bp]	Calibrated Conc. [ng/µl]	Assigned Conc. [ng/µl]	Peak Molarity [nmol/l]	% Integrated Area	Peak Comment	Observations
25	5.04	-	310	-		Lower Marker
590	70.5	-	184	100.00		
1500	6.50	6.50	6.67	-		Upper Marker

© Agilent Technologies, Inc. 2019

C1: #27 T2

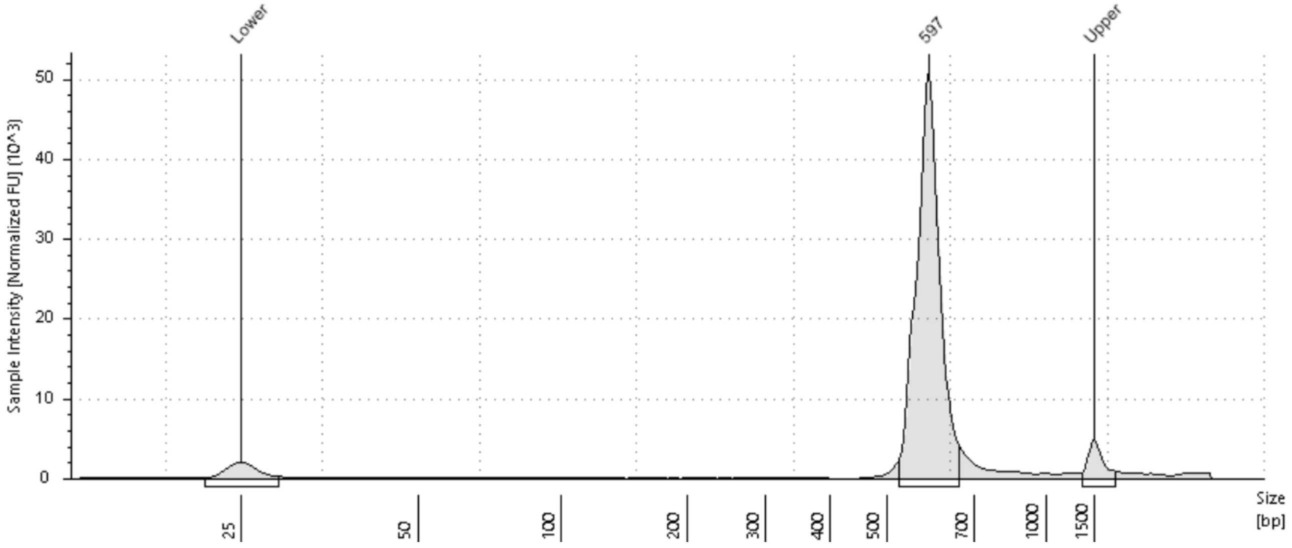

Sample Table

Well	Conc. [ng/µl]	Sample Description	Alert	Observations
C1	109	#27 T2		

Peak Table

Size [bp]	Calibrated Conc. [ng/µl]	Assigned Conc. [ng/µl]	Peak Molarity [nmol/l]	% Integrated Area	Peak Comment	Observations
25	5.81	-	358	-		Lower Marker
597	109	-	280	100.00		
1500	6.50	6.50	6.67	-		Upper Marker

© Agilent Technologies, Inc. 2019

D1: #44 T2

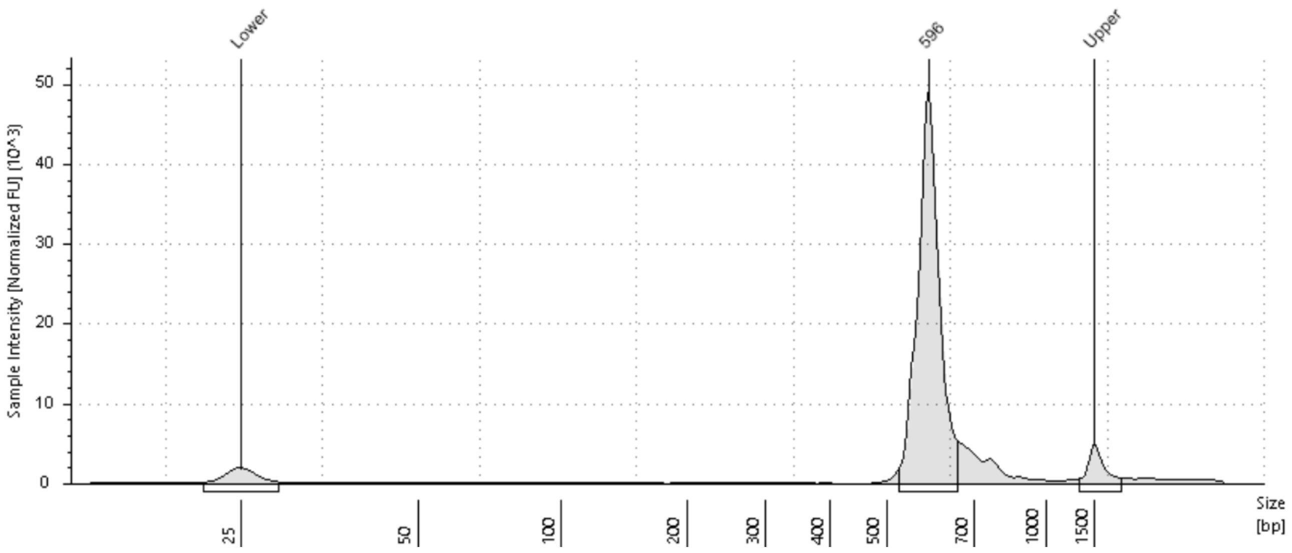

Sample Table

Well	Conc. [ng/µl]	Sample Description	Alert	Observations
D1	90.1	#44 T2		

Peak Table

Size [bp]	Calibrated Conc. [ng/µl]	Assigned Conc. [ng/µl]	Peak Molarity [nmol/l]	% Integrated Area	Peak Comment	Observations
25	5.28	-	325	-		Lower Marker
596	90.1	-	232	100.00		
1500	6.50	6.50	6.67	-		Upper Marker

© Agilent Technologies, Inc. 2019

E1: #9 T2

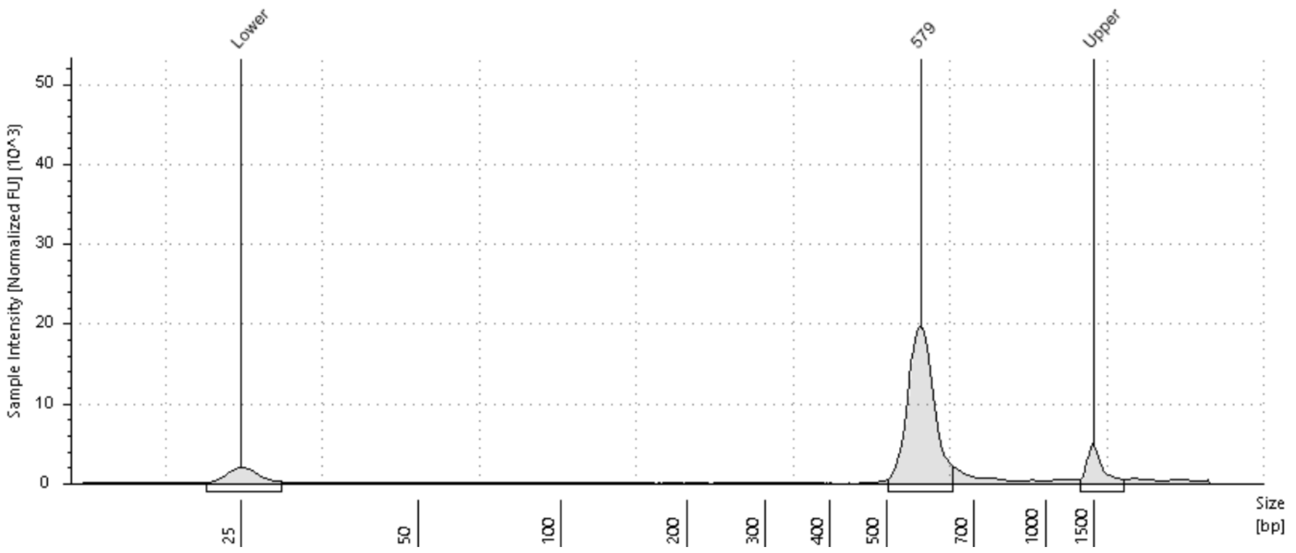

Sample Table

Well	Conc. [ng/µl]	Sample Description	Alert	Observations
E1	45.4	#9 T2		

Peak Table

Size [bp]	Calibrated Conc. [ng/µl]	Assigned Conc. [ng/µl]	Peak Molarity [nmol/l]	% Integrated Area	Peak Comment	Observations
25	5.61	-	345	-		Lower Marker
579	45.4	-	120	100.00		
1500	6.50	6.50	6.67	-		Upper Marker

© Agilent Technologies, Inc. 2019

F1: #10 T2

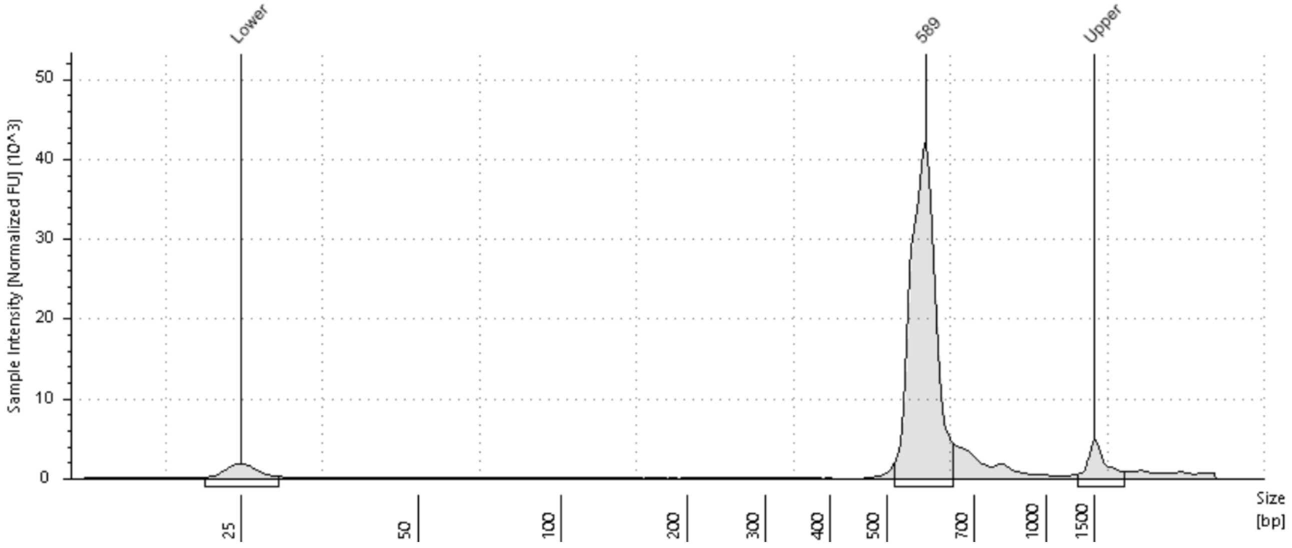

Sample Table

Well	Conc. [ng/µl]	Sample Description	Alert	Observations
F1	82.1	#10 T2		

Peak Table

Size [bp]	Calibrated Conc. [ng/µl]	Assigned Conc. [ng/µl]	Peak Molarity [nmol/l]	% Integrated Area	Peak Comment	Observations
25	4.70	-	290	-		Lower Marker
589	82.1	-	214	100.00		
1500	6.50	6.50	6.67	-		Upper Marker

© Agilent Technologies, Inc. 2019

G1: #8 T2

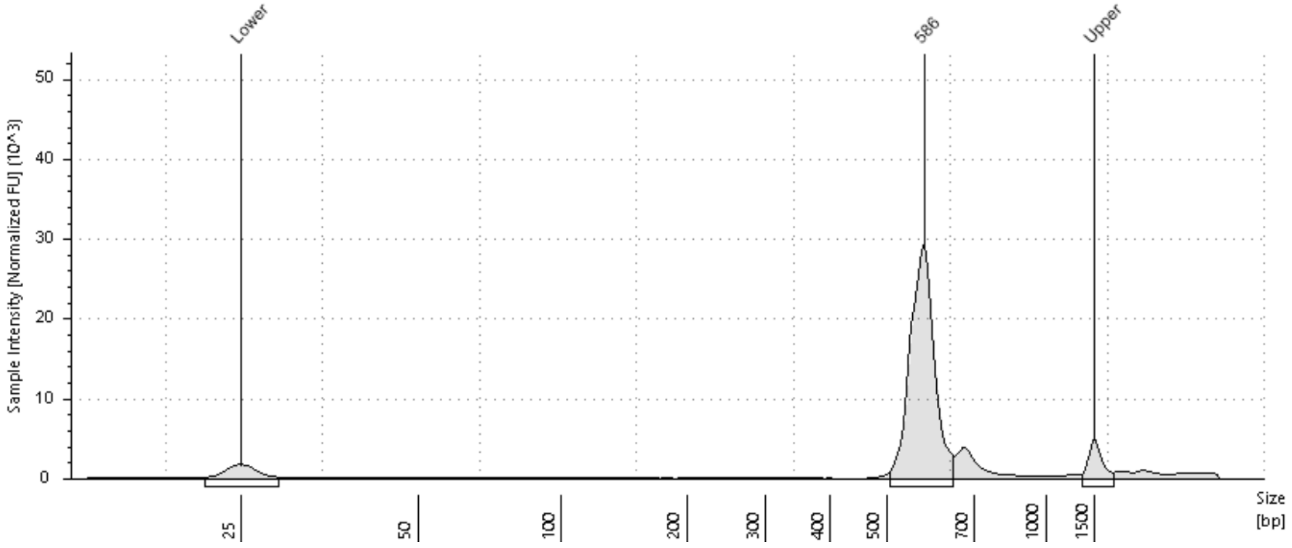

Sample Table

Well	Conc. [ng/µl]	Sample Description	Alert	Observations
Gl	70.9	#8 T2		

Peak Table

Size [bp]	Calibrated Conc. [ng/µl]	Assigned Conc. [ng/µl]	Peak Molarity [nmol/l]	% Integrated Area	Peak Comment	Observations
25	5.56	-	342	-		Lower Marker
586	70.9	-	186	100.00		
1500	6.50	6.50	6.67	-		Upper Marker

© Agilent Technologies, Inc. 2019

H1: #17 T2

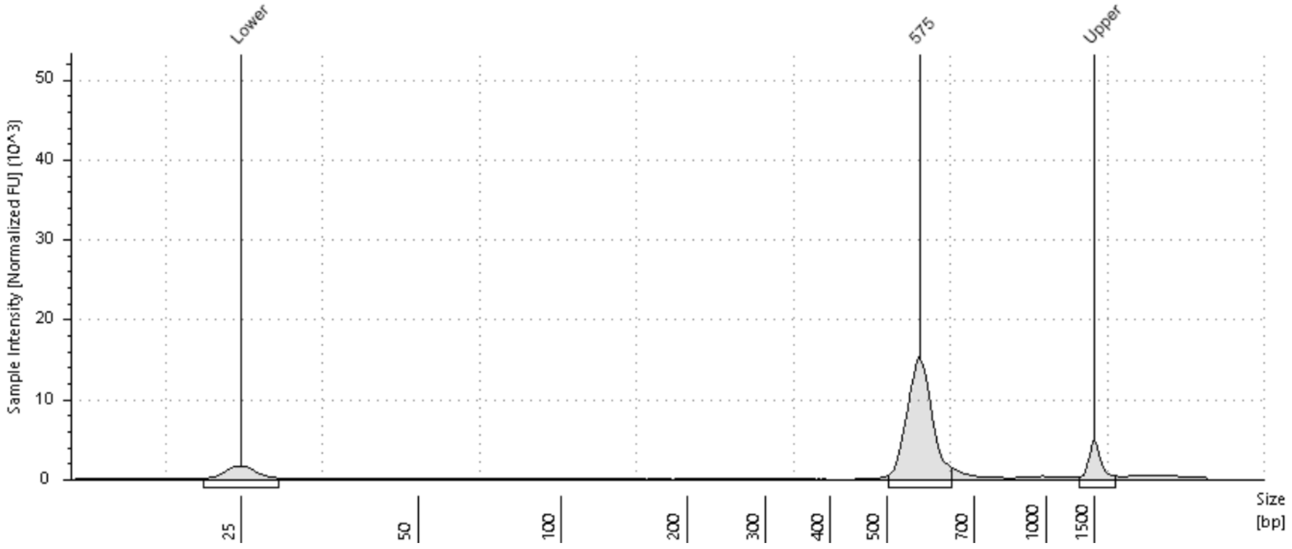

Sample Table

Well	Conc. [ng/µl]	Sample Description	Alert	Observations
H1	41.1	#17 T2		

Peak Table

Size [bp]	Calibrated Conc. [ng/µl]	Assigned Conc. [ng/µl]	Peak Molarity [nmol/l]	% Integrated Area	Peak Comment	Observations
25	5.76	-	355	-		Lower Marker
575	41.1	-	110	100.00		
1500	6.50	6.50	6.67	-		edited Upper Marker

© Agilent Technologies, Inc. 2019

A2: #35 T2

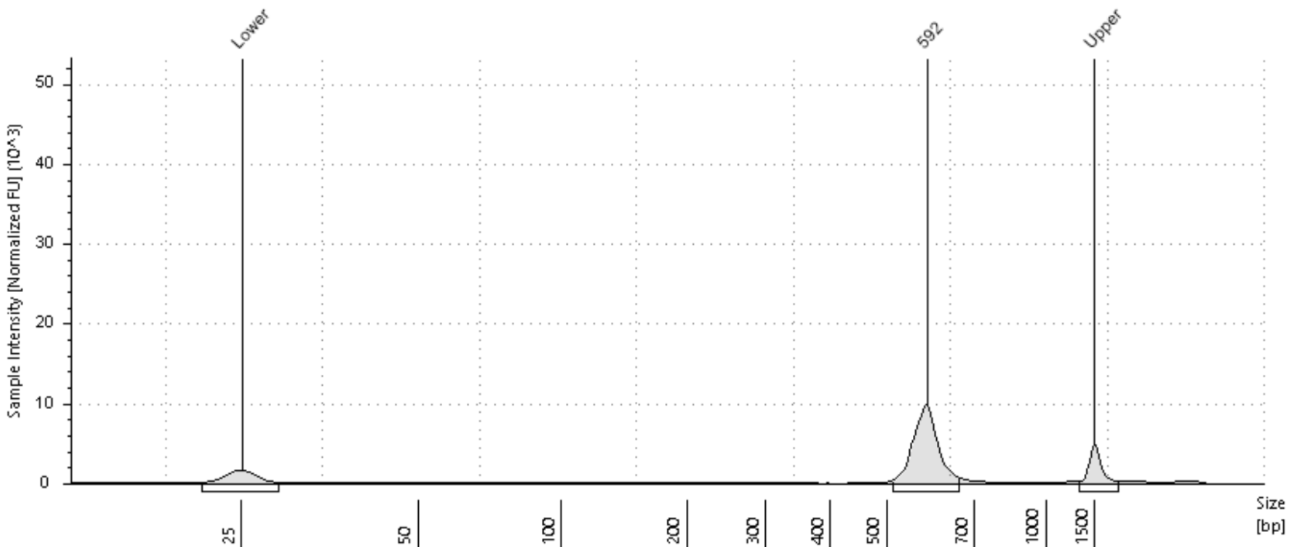

Sample Table

Well	Conc. [ng/µl]	Sample Description	Alert	Observations
A2	26.5	#35 T2		

Peak Table

Size [bp]	Calibrated Conc. [ng/µl]	Assigned Conc. [ng/µl]	Peak Molarity [nmol/l]	% Integrated Area	Peak Comment	Observations
25	5.69	-	350	-		Lower Marker
592	26.5	-	69.0	100.00		
1500	6.50	6.50	6.67	-		edited Upper Marker

 © Agilent Technologies, Inc. 2019

B2: #15 T2

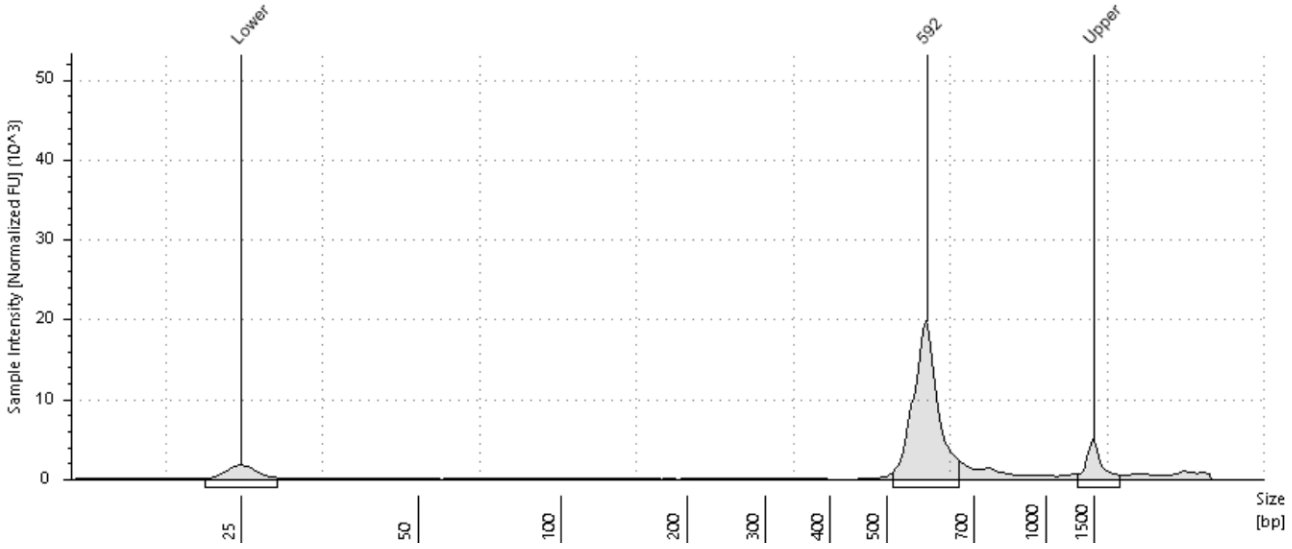

Sample Table

Well	Conc. [ng/µl]	Sample Description	Alert	Observations
B2	45.5	#15 T2		

Peak Table

Size [bp]	Calibrated Conc. [ng/µl]	Assigned Conc. [ng/µl]	Peak Molarity [nmol/l]	% Integrated Area	Peak Comment	Observations
25	5.07	-	312	-		Lower Marker
592	45.5	-	118	100.00		
1500	6.50	6.50	6.67	-		edited Upper Marker

© Agilent Technologies, Inc. 2019

C2: #6 T2

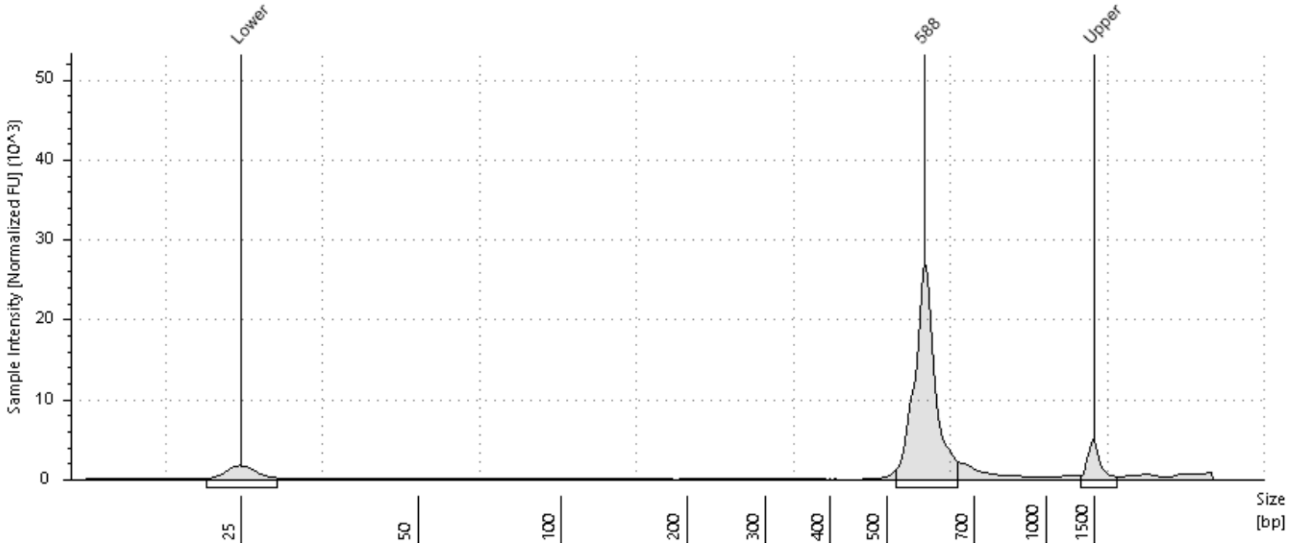

Sample Table

Well	Conc. [ng/µl]	Sample Description	Alert	Observations
C2	59.8	#6 T2		

Peak Table

Size [bp]	Calibrated Conc. [ng/µl]	Assigned Conc. [ng/µl]	Peak Molarity [nmol/l]	% Integrated Area	Peak Comment	Observations
25	5.69	-	350	-		Lower Marker
588	59.8	-	156	100.00		
1500	6.50	6.50	6.67	-		Upper Marker

© Agilent Technologies, Inc. 2019

D2: #13 T2

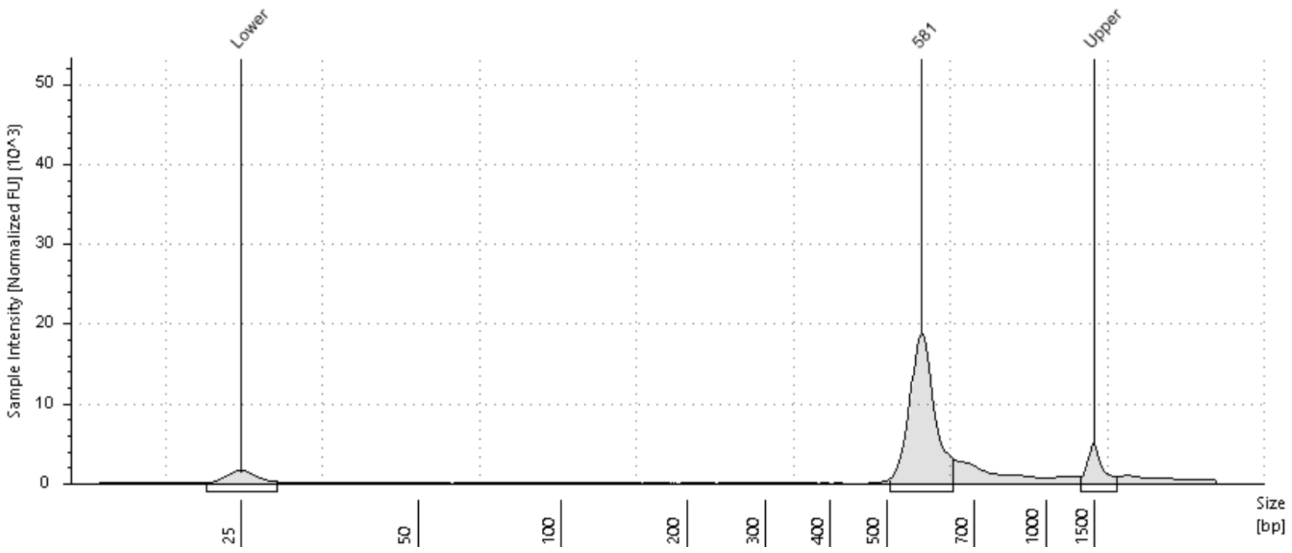

Sample Table

Well	Conc. [ng/µl]	Sample Description	Alert	Observations
D2	43.6	#13 T2		

Peak Table

Size [bp]	Calibrated Conc. [ng/µl]	Assigned Conc. [ng/µl]	Peak Molarity [nmol/l]	% Integrated Area	Peak Comment	Observations
25	4.57	-	281	-		Lower Marker
581	43.6	-	116	100.00		
1500	6.50	6.50	6.67	-		edited Upper Marker

© Agilent Technologies, Inc. 2019

E2: #32 T2

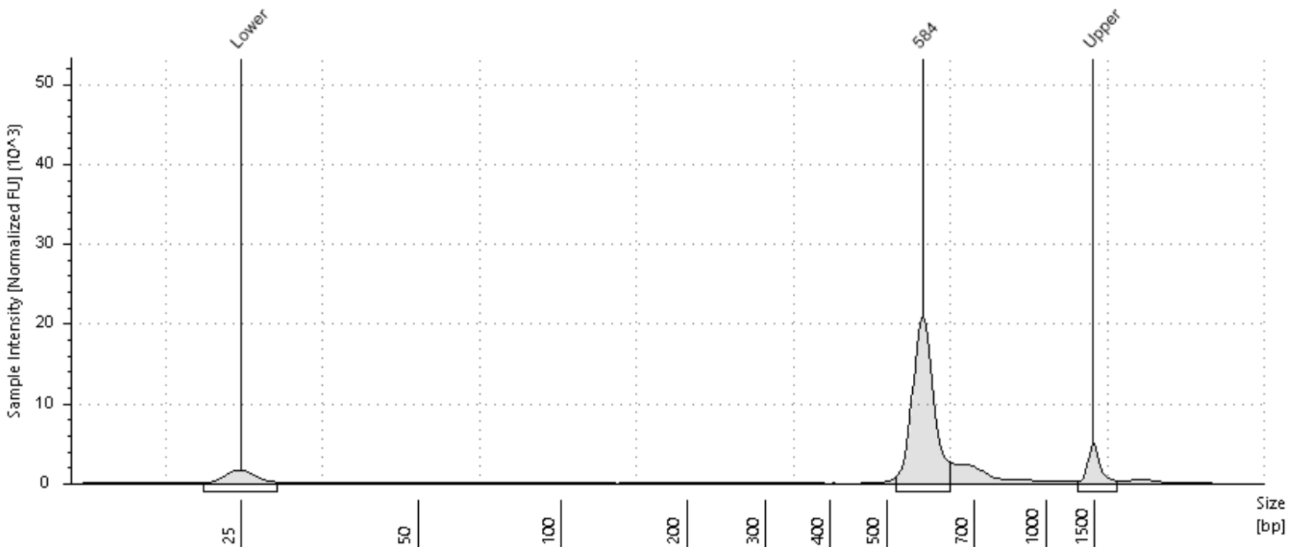

Sample Table

Well	Conc. [ng/µl]	Sample Description	Alert	Observations
E2	48.1	#32 T2		

Peak Table

Size [bp]	Calibrated Conc. [ng/µl]	Assigned Conc. [ng/µl]	Peak Molarity [nmol/l]	% Integrated Area	Peak Comment	Observations
25	5.52	-	340	-		Lower Marker
584	48.1	-	127	100.00		
1500	6.50	6.50	6.67	-		Upper Marker

 © Agilent Technologies, Inc. 2019

F2: #14 T2

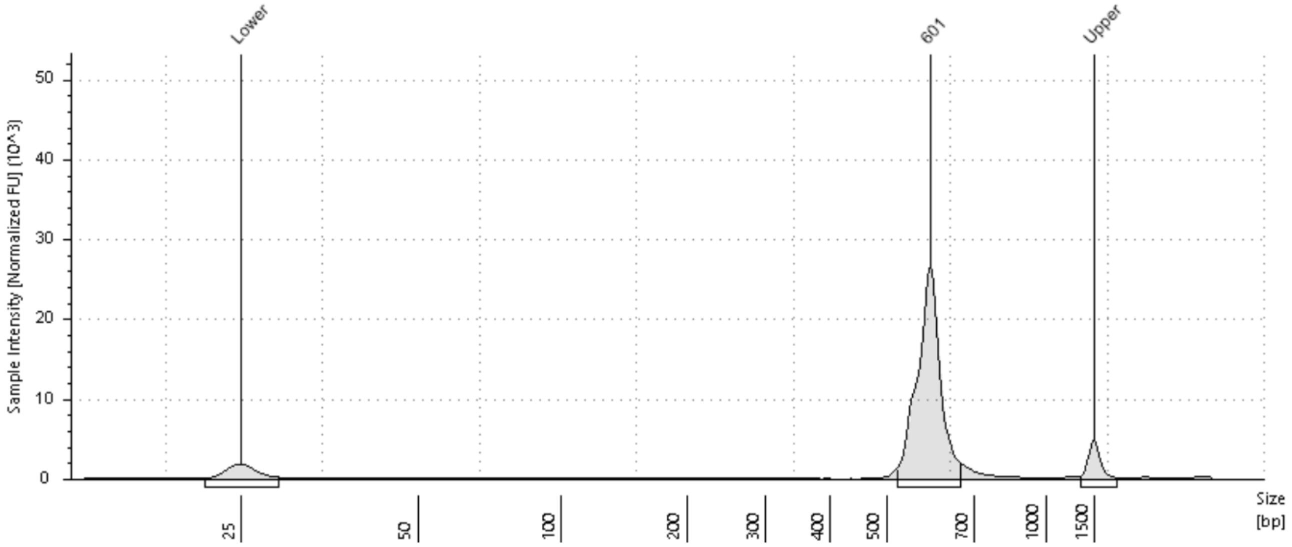

Sample Table

Well	Conc. [ng/µl]	Sample Description	Alert	Observations
F2	68.3	#14 T2		

Peak Table

Size [bp]	Calibrated Conc. [ng/µl]	Assigned Conc. [ng/µl]	Peak Molarity [nmol/l]	% Integrated Area	Peak Comment	Observations
25	6.42	-	395	-		Lower Marker
601	68.3	-	175	100.00		
1500	6.50	6.50	6.67	-		Upper Marker

© Agilent Technologies, Inc. 2019

G2: #60 T2

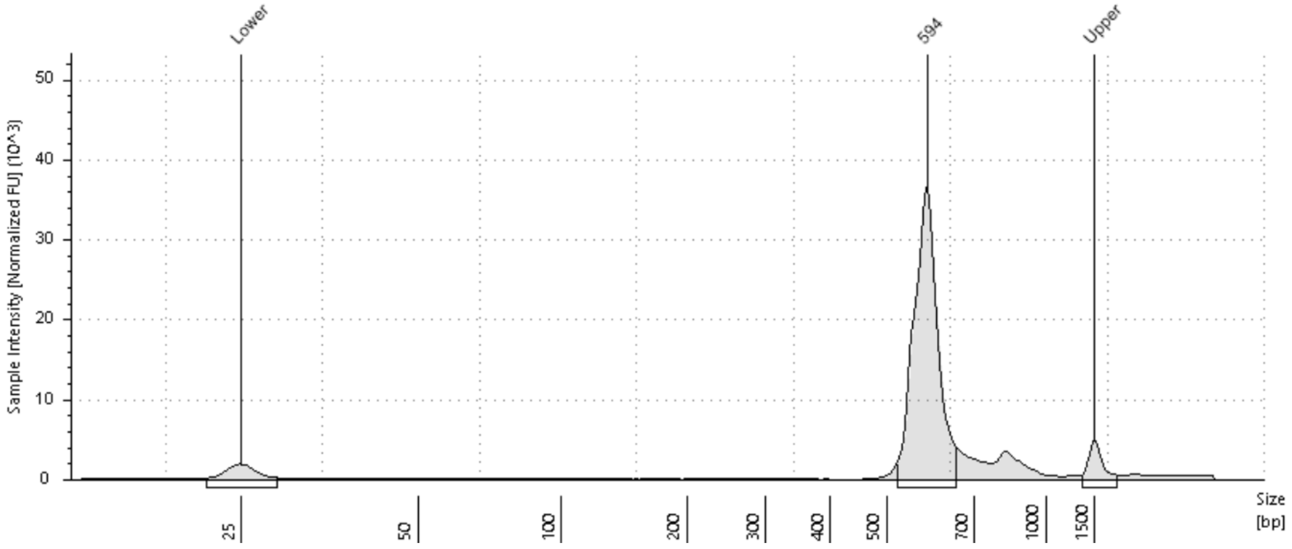

Sample Table

Well	Conc. [ng/µl]	Sample Description	Alert	Observations
G2	86.8	#60 T2		

Peak Table

Size [bp]	Calibrated Conc. [ng/µl]	Assigned Conc. [ng/µl]	Peak Molarity [nmol/l]	% Integrated Area	Peak Comment	Observations
25	5.70	-	351	-		Lower Marker
594	86.8	-	225	100.00		
1500	6.50	6.50	6.67	-		Upper Marker

© Agilent Technologies, Inc. 2019

H2: #23 T2

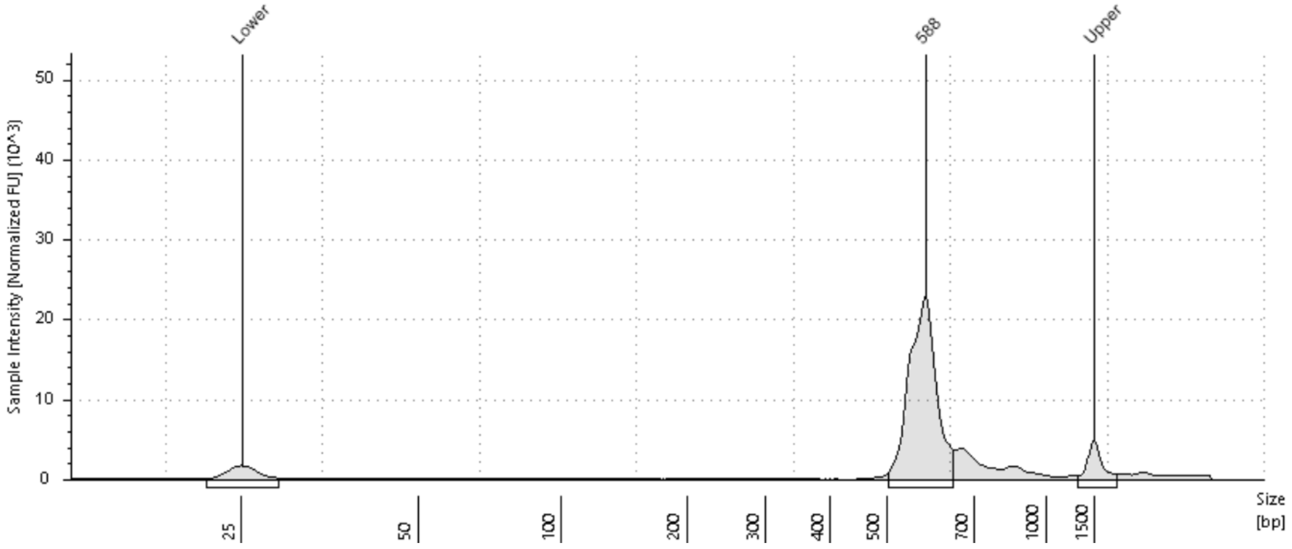

Sample Table

Well	Conc. [ng/µl]	Sample Description	Alert	Observations
H2	60.3	#23 T2		

Peak Table

Size [bp]	Calibrated Conc. [ng/µl]	Assigned Conc. [ng/µl]	Peak Molarity [nmol/l]	% Integrated Area	Peak Comment	Observations
25	5.05	-	311	-		Lower Marker
588	60.3	-	158	100.00		
1500	6.50	6.50	6.67	-		Upper Marker

© Agilent Technologies, Inc. 2019

A3: #12 T2

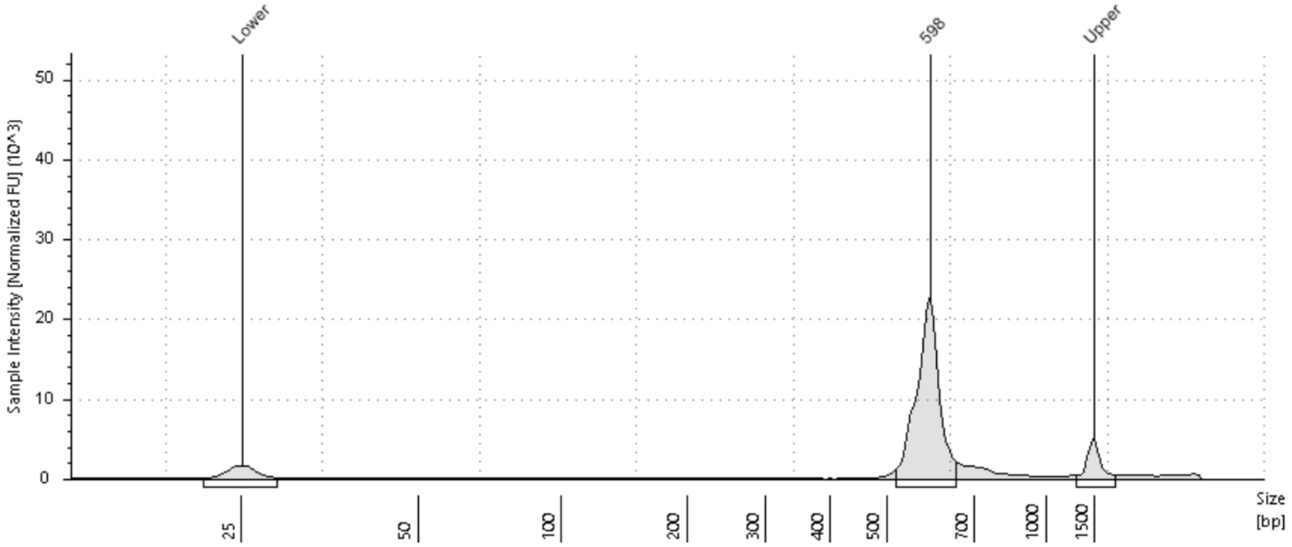

Sample Table

Well	Conc. [ng/µl]	Sample Description	Alert	Observations
A3	51.8	#12 T2		

Peak Table

Size [bp]	Calibrated Conc. [ng/µl]	Assigned Conc. [ng/µl]	Peak Molarity [nmol/l]	% Integrated Area	Peak Comment	Observations
25	5.13	-	316	-		Lower Marker
598	51.8	-	133	100.00		
1500	6.50	6.50	6.67	-		edited Upper Marker

© Agilent Technologies, Inc. 2019

B3: #34 T2

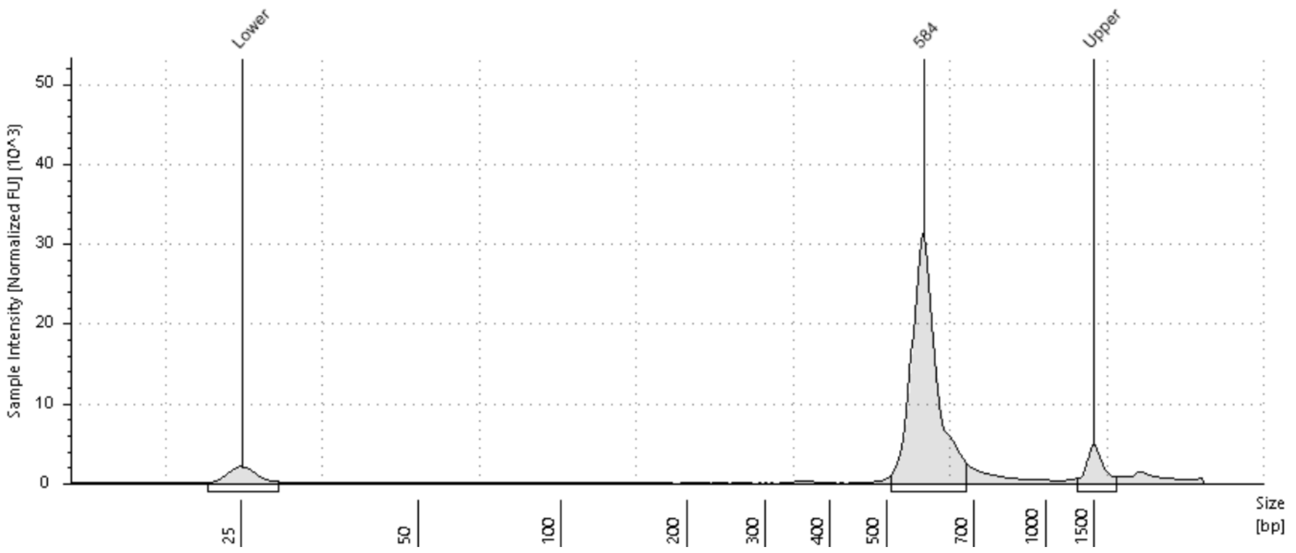

Sample Table

Well	Conc. [ng/µl]	Sample Description	Alert	Observations
B3	65.3	#34 T2		

Peak Table

Size [bp]	Calibrated Conc. [ng/µl]	Assigned Conc. [ng/µl]	Peak Molarity [nmol/l]	% Integrated Area	Peak Comment	Observations
25	5.16	-	318	-		Lower Marker
584	65.3	-	172	100.00		
1500	6.50	6.50	6.67	-		edited Upper Marker

© Agilent Technologies, Inc. 2019

C3: #30 T2

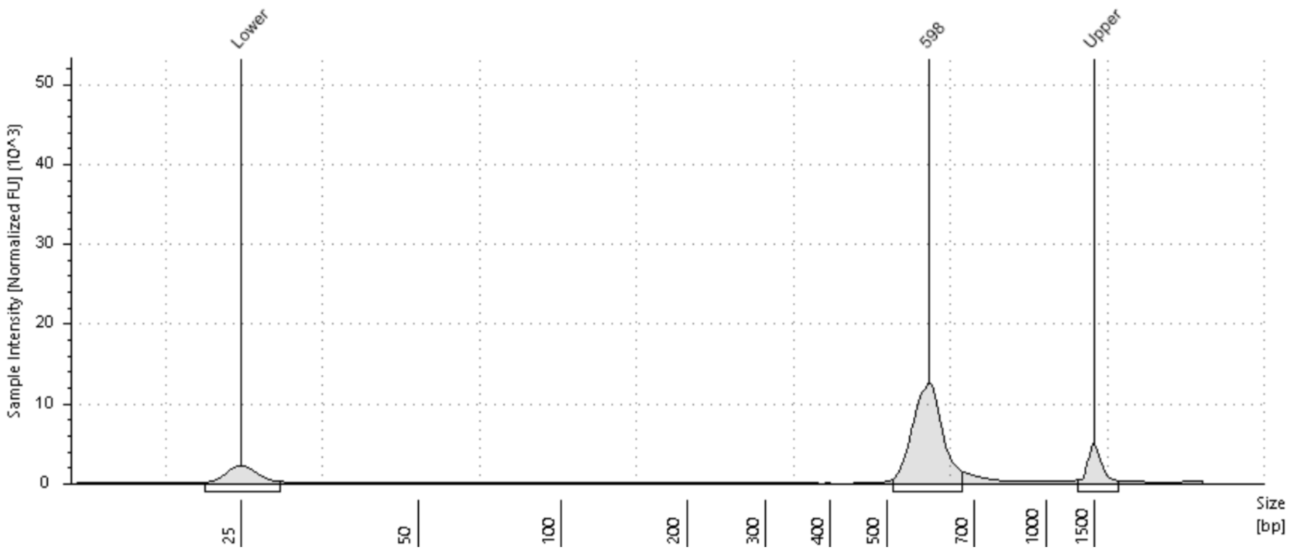

Sample Table

Well	Conc. [ng/µl]	Sample Description	Alert	Observations
C3	35.4	#30 T2		

Peak Table

Size [bp]	Calibrated Conc. [ng/µl]	Assigned Conc. [ng/µl]	Peak Molarity [nmol/l]	% Integrated Area	Peak Comment	Observations
25	6.62	-	407	-		Lower Marker
598	35.4	-	90.9	100.00		
1500	6.50	6.50	6.67	-		Upper Marker

© Agilent Technologies, Inc. 2019

D3: #21 T2

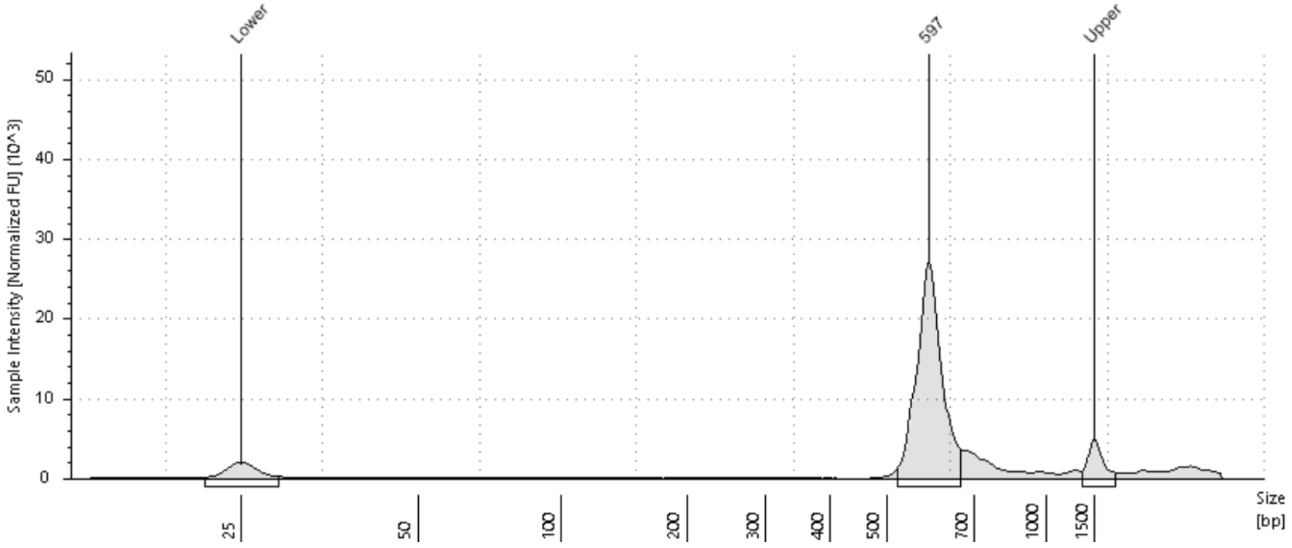

Sample Table

Well	Conc. [ng/µl]	Sample Description	Alert	Observations
D3	62.4	#21 T2		

Peak Table

Size [bp]	Calibrated Conc. [ng/µl]	Assigned Conc. [ng/µl]	Peak Molarity [nmol/l]	% Integrated Area	Peak Comment	Observations
25	5.72	-	352	-		Lower Marker
597	62.4	-	161	100.00		
1500	6.50	6.50	6.67	-		edited Upper Marker

© Agilent Technologies, Inc. 2019

E3: #28 T2

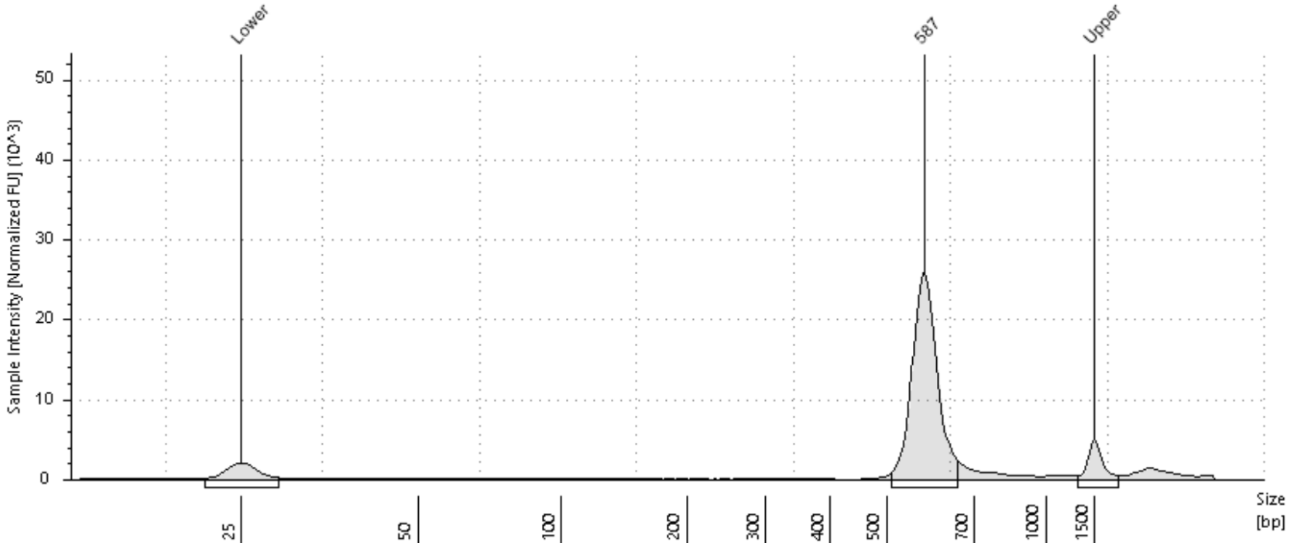

Sample Table

Well	Conc. [ng/µl]	Sample Description	Alert	Observations
E3	60.9	#28 T2		

Peak Table

Size [bp]	Calibrated Conc. [ng/µl]	Assigned Conc. [ng/µl]	Peak Molarity [nmol/l]	% Integrated Area	Peak Comment	Observations
25	5.94	-	366	-		Lower Marker
587	60.9	-	159	100.00		
1500	6.50	6.50	6.67	-		edited Upper Marker

© Agilent Technologies, Inc. 2019

F3: #50 T2

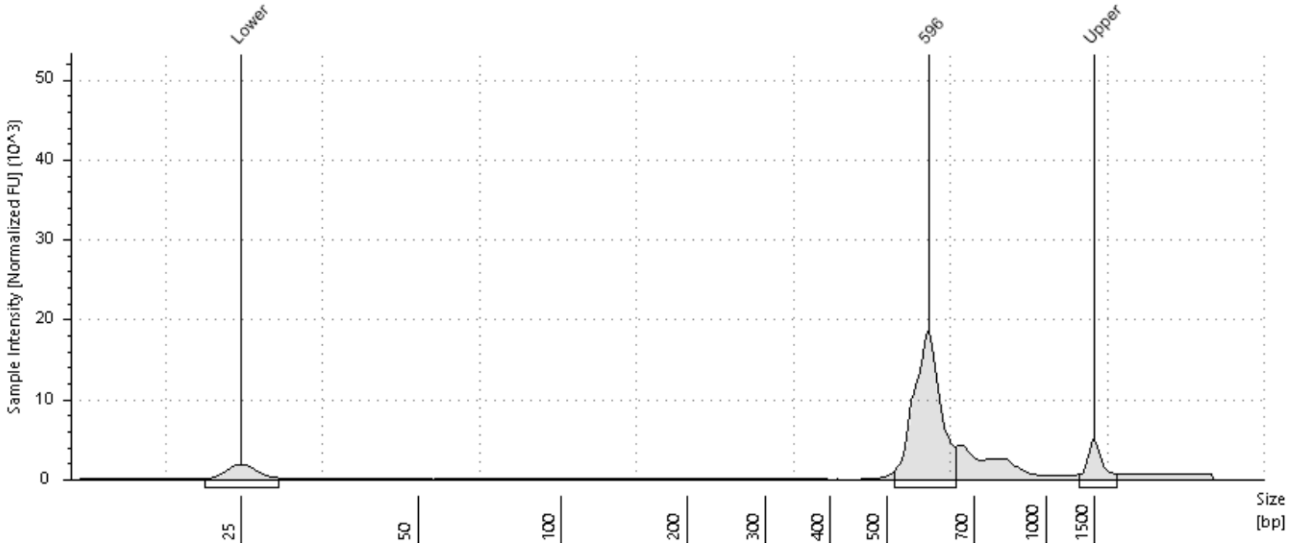

Sample Table

Well	Conc. [ng/µl]	Sample Description	Alert	Observations
F3	46.0	#50 T2		

Peak Table

Size [bp]	Calibrated Conc. [ng/µl]	Assigned Conc. [ng/µl]	Peak Molarity [nmol/l]	% Integrated Area	Peak Comment	Observations
25	5.50	-	338	-		Lower Marker
596	46.0	-	119	100.00		
1500	6.50	6.50	6.67	-		Upper Marker

© Agilent Technologies, Inc. 2019

G3: #40 T2

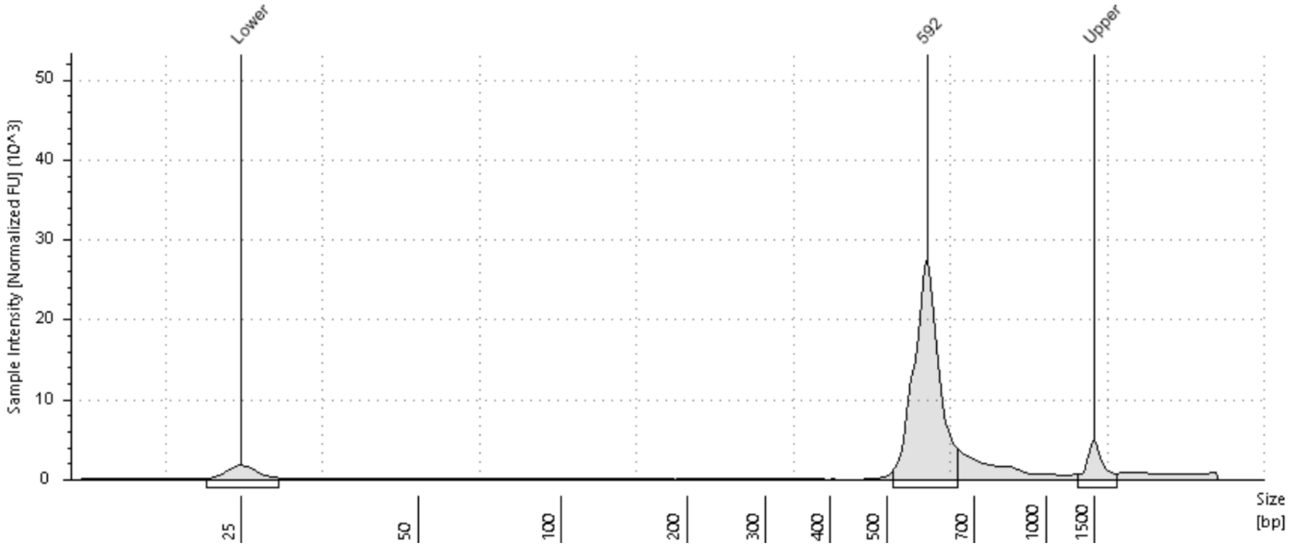

Sample Table

Well	Conc. [ng/µl]	Sample Description	Alert	Observations
G3	63.7	#40 T2		

Peak Table

Size [bp]	Calibrated Conc. [ng/µl]	Assigned Conc. [ng/µl]	Peak Molarity [nmol/l]	% Integrated Area	Peak Comment	Observations
25	5.03	-	309	-		Lower Marker
592	63.7	-	165	100.00		
1500	6.50	6.50	6.67	-		Upper Marker

© Agilent Technologies, Inc. 2019

H3: #55 T2

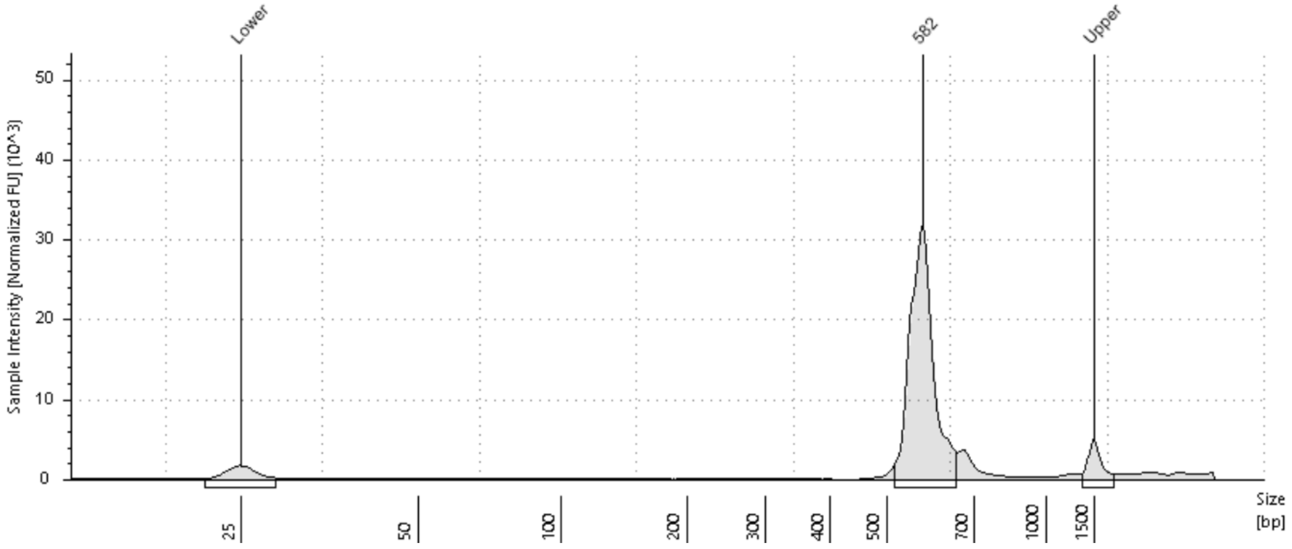

Sample Table

Well	Conc. [ng/µl]	Sample Description	Alert	Observations
H3	76.0	#55 T2		

Peak Table

Size [bp]	Calibrated Conc. [ng/µl]	Assigned Conc. [ng/µl]	Peak Molarity [nmol/l]	% Integrated Area	Peak Comment	Observations
25	5.29	-	325	-		Lower Marker
582	76.0	-	201	100.00		
1500	6.50	6.50	6.67	-		Upper Marker

© Agilent Technologies, Inc. 2019

A4: #41 T2

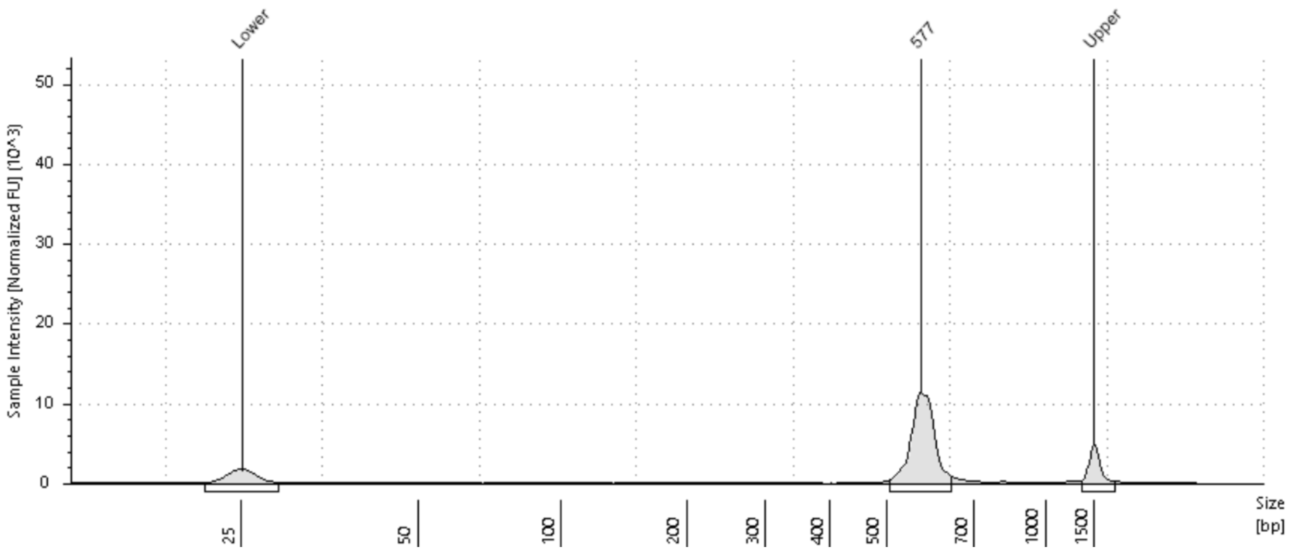

Sample Table

Well	Conc. [ng/µl]	Sample Description	Alert	Observations
A4	31.7	#41 T2		

Peak Table

Size [bp]	Calibrated Conc. [ng/µl]	Assigned Conc. [ng/µl]	Peak Molarity [nmol/l]	% Integrated Area	Peak Comment	Observations
25	6.30	-	388	-		Lower Marker
577	31.7	-	84.5	100.00		
1500	6.50	6.50	6.67	-		Upper Marker

B4: #46 T2

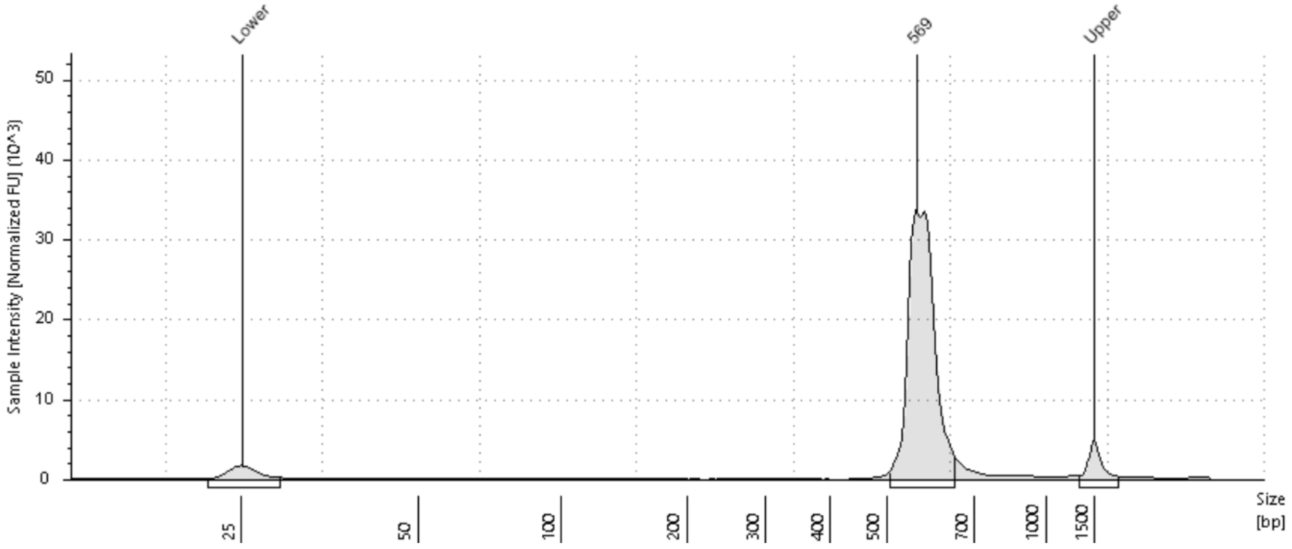

Sample Table

Well	Conc. [ng/µl]	Sample Description	Alert	Observations
B4	93.5	#46 T2		

Peak Table

Size [bp]	Calibrated Conc. [ng/µl]	Assigned Conc. [ng/µl]	Peak Molarity [nmol/l]	% Integrated Area	Peak Comment	Observations
25	5.28	-	325	-		Lower Marker
569	93.5	-	253	100.00		
1500	6.50	6.50	6.67	-		Upper Marker

© Agilent Technologies, Inc. 2019

C4: #2 T2

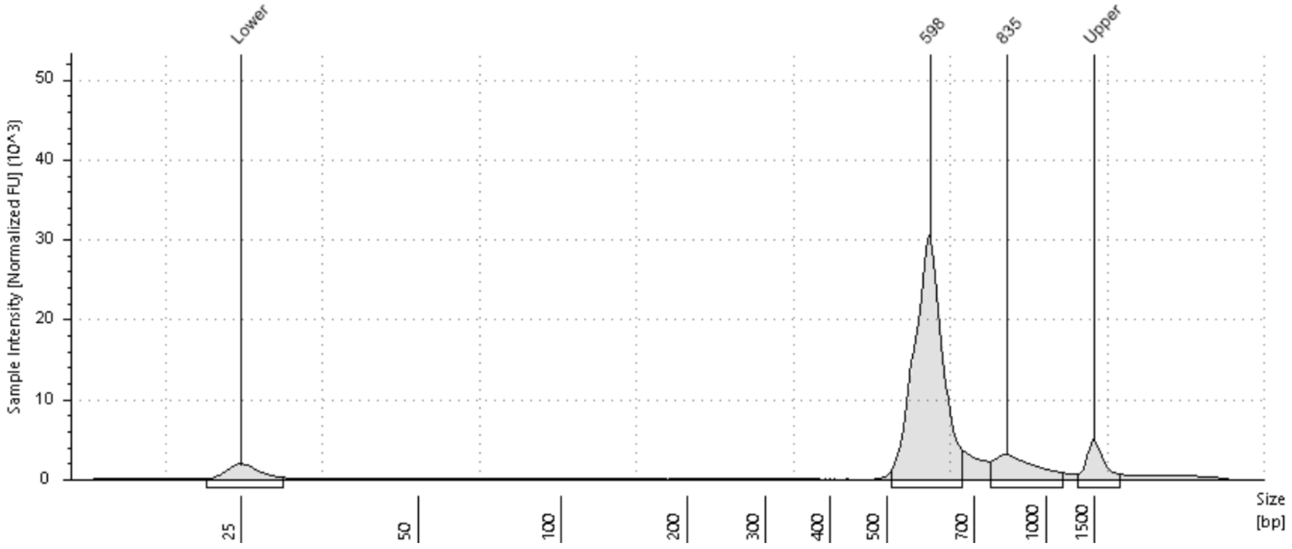

Sample Table

Well	Conc. [ng/µl]	Sample Description	Alert	Observations
C4	77.1	#2 T2		

Peak Table

Size [bp]	Calibrated Conc. [ng/µl]	Assigned Conc. [ng/µl]	Peak Molarity [nmol/l]	% Integrated Area	Peak Comment	Observations
25	4.85	-	299	-		Lower Marker
598	67.1	-	173	87.04		
835	9.99	-	18.4	12.96		
1500	6.50	6.50	6.67	-		Upper Marker

© Agilent Technologies, Inc. 2019

D4: #49 T2

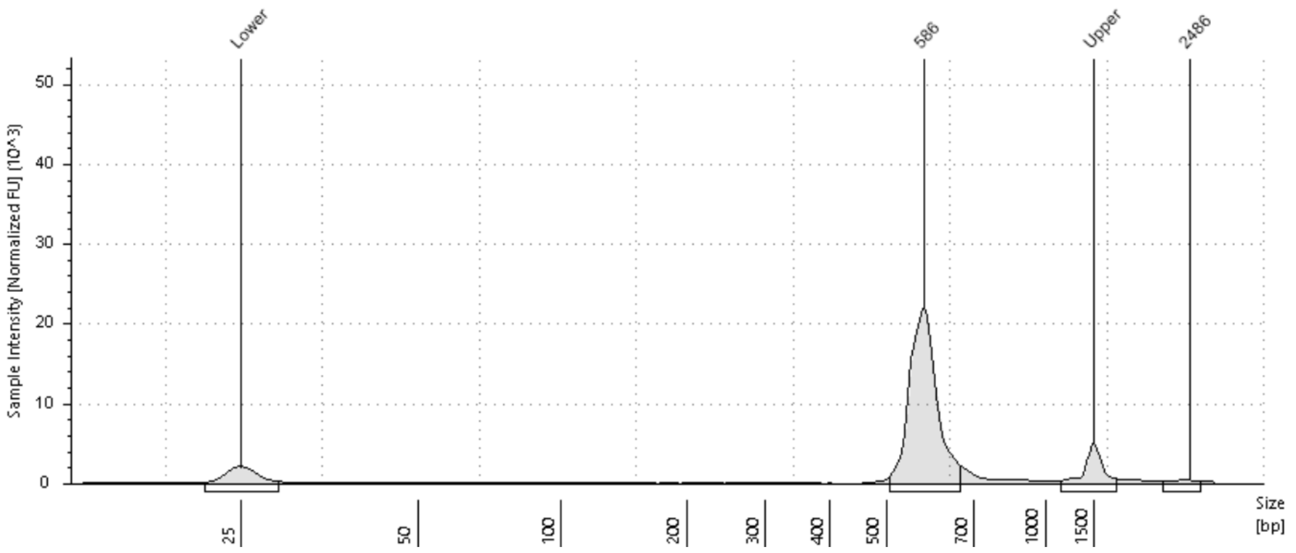

Sample Table

Well	Conc. [ng/µl]	Sample Description	Alert	Observations
D4	47.8	#49 T2	⚠	Peak out of Sizing Range

Peak Table

Size [bp]	Calibrated Conc. [ng/µl]	Assigned Conc. [ng/µl]	Peak Molarity [nmol/l]	% Integrated Area	Peak Comment	Observations
25	5.24	-	322	-		Lower Marker
586	46.9	-	123	98.20		
1500	6.50	6.50	6.67	-		Upper Marker
2486	0.860	-	0.532	1.80		Peak outside of Sizing Range

© Agilent Technologies, Inc. 2019

E4: #29 T2

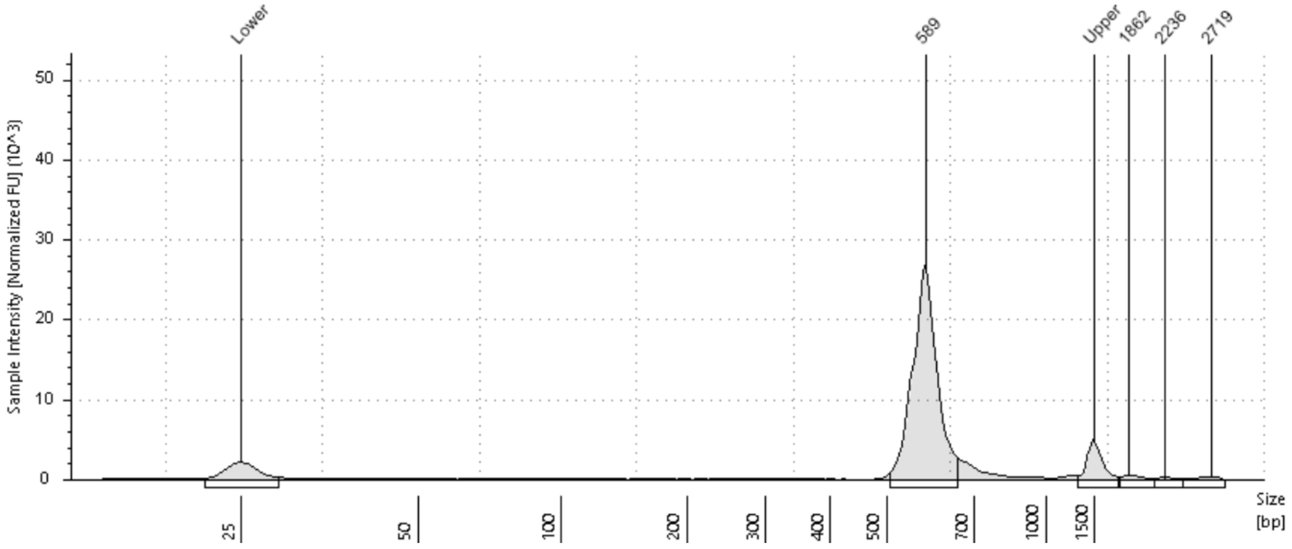

Sample Table

Well	Conc. [ng/µl]	Sample Description	Alert	Observations
E4	61.2	#29 T2	⚠	Peak out of Sizing Range

Peak Table

Size [bp]	Calibrated Conc. [ng/µl]	Assigned Conc. [ng/µl]	Peak Molarity [nmol/l]	% Integrated Area	Peak Comment	Observations
25	6.17	-	380	-		Lower Marker
589	59.4	-	155	97.02		
1500	6.50	6.50	6.67	-		Upper Marker
1862	0.846	-	0.699	1.38		Peak outside of Sizing Range
2236	0.347	-	0.239	0.57		Peak outside of Sizing Range
2719	0.629	-	0.356	1.03		Peak outside of Sizing Range

© Agilent Technologies, Inc. 2019

C10: CTRL- H2O

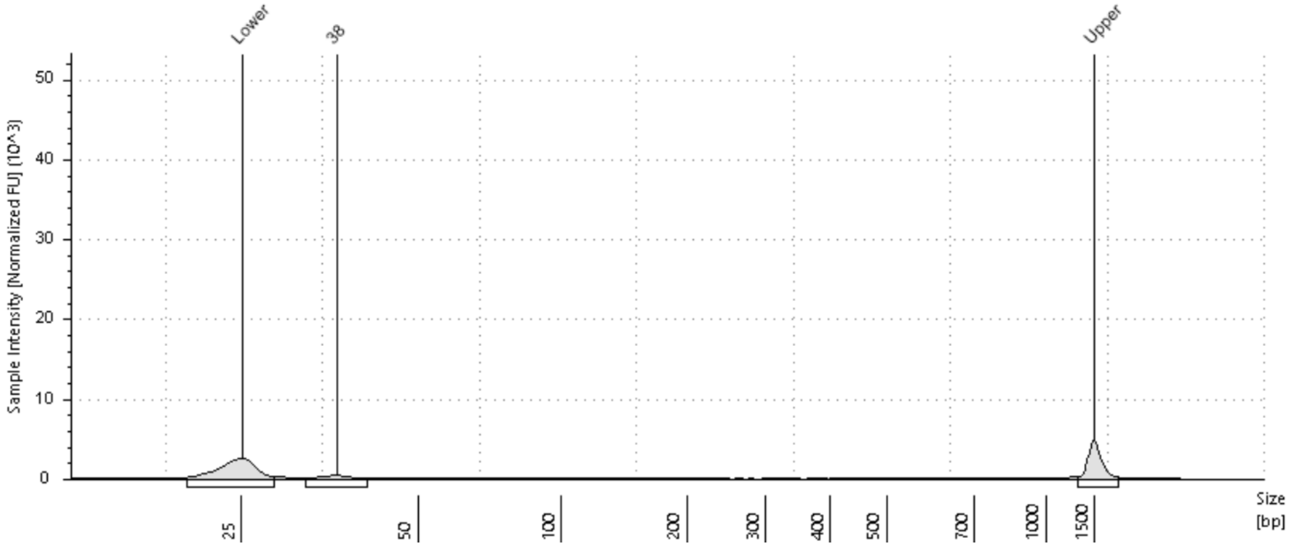

Sample Table

Well	Conc. [ng/µl]	Sample Description	Alert	Observations
C10	1.25	CTRL- H2O		

Peak Table

Size [bp]	Calibrated Conc. [ng/µl]	Assigned Conc. [ng/µl]	Peak Molarity [nmol/l]	% Integrated Area	Peak Comment	Observations
25	9.25	-	569	-		Lower Marker
38	1.25	-	50.1	100.00		
1500	6.50	6.50	6.67	-		Upper Marker

© Agilent Technologies, Inc. 2019
