# Supplementary material for: Early Life Microbiota Colonization at Six Months of Age: A Transitional Time Point
Source: Front Cell Infect Microbiol. 2021 Mar 26;11:590202. doi: 10.3389/fcimb.2021.590202 (PMC8032992; doi:10.3389/fcimb.2021.590202)
Supplement: Supplementary file 4 [file DataSheet_4.docx]

Supplementary Material

**Supplementary Figure 1.** Principal components analysis (PCA). The PCA was performed using genera with a median relative abundance (16S rRNA metagenetics) >0.1%. Panel A shows PCA of all T2 fecal samples (infants at six months of age). Panel B biplot of individuals and variables calculated by using factoextra R package. The factoextra cos2 calculated PCA values shows the importance of a principal component for a given observation (vector of original variables). Components with a large value of cos2 contribute to a relatively large portion of the total distance.

**Supplementary Figure 2.** Discriminant analysis of principal components (DAPC) plus cluster assignment. The DAPC was performed using genera with a median relative abundance (16S rRNA metagenetics) >0.1% and without superimposing any sample membership. In DAPC scoreplot each of the T2- fecal samples (gut microbiota of infants at six months of age) of the four clusters (1, 2, 3, and 4) has a different point shape and color. The table on the right reports DAPC sample assignment to each cluster.
