# Supplementary material for: Early Life Microbiota Colonization at Six Months of Age: A Transitional Time Point
Source: Front Cell Infect Microbiol. 2021 Mar 26;11:590202. doi: 10.3389/fcimb.2021.590202 (PMC8032992; doi:10.3389/fcimb.2021.590202)
Supplement: Supplementary file 6 [file Table_1.docx]

**Tab. 1S**. General characteristics of the dyads at T2. Data are presented as Average, Median and Standard Deviation (SD)

| Dyad | Parameters | Average | Median | SD |
| --- | --- | --- | --- | --- |
| Mothers (*n* = 53) | **Age** (years) | 33 | 34 | 5 |
|  | **Pre- pregnancy weight** (kg) | 61.98 | 60 | 0.71 |
|  |  |  |  |  |
|  |  |  |  |  |
|  | **Pre -pregnancy height (cm)** | 164.54 | 164.0 | 4.95 |
|  |  |  |  |  |
|  |  |  |  |  |
|  | **Pre-pregnancy BMI** (Kg/m^2^) | 22.76 | 22 | 1.41 |
|  |  |  |  |  |
|  |  |  |  |  |
|  | **Weight gain during pregnancy** (Kg) ^a^ | 12.67 | 12,5 | 0.71 |
|  |  |  |  |  |
|  |  |  |  |  |
| Infants  *(n=53; M=28; F=25)* | **Gestational age at birth** (weeks) | 39.4 | 40 | 0 |
|  |  |  |  |  |
|  |  |  |  |  |
|  | **Birth weight (T0)** (Kg) | 3.12 | 3,05 | 0.46 |
|  |  |  |  |  |
|  |  |  |  |  |
|  | **T2 Weight** (Kg) | 7.29 | 7,33 | 0.46 |
|  |  |  |  |  |
|  |  |  |  |  |
|  | **T2 Length** (cm) | 66.55 | 66 | 0.71 |
|  |  |  |  |  |
|  | **T2 Cranial circumference** (cm) | 42.91 | 43 | 1.41 |

^a^ according to the Institute of Medicine (US), guidelines [Institute of Medicine, US; Weight gain during pregnancy: re-examining the guidelines. Washington, DC. National Academies Press; National Academy of Sciences]
